# *Ex-vivo* culture of human hypertrophic cardiomyopathy hearts: Functional and metabolic changes during long-term culture

**DOI:** 10.1016/j.isci.2026.115308

**Published:** 2026-03-11

**Authors:** Ali Nassar, Vincent A.J. Warnaar, Inez Duursma, Bauke V. Schomakers, Chahida Chaami, Julien Ochala, Michel van Weeghel, Riekelt H. Houtkooper, Michelle Michels, Andreas Dendorfer, Diederik W.D. Kuster, Jolanda van der Velden

**Affiliations:** 1 Department of Physiology, Amsterdam Cardiovascular Sciences, Amsterdam University Medical Centre (UMC), Amsterdam, the Netherlands; 2 Laboratory Genetic Metabolic Diseases, Amsterdam UMC, Location University of Amsterdam, Amsterdam, the Netherlands; 3 Department of Biomedical Sciences, University of Copenhagen, Copenhagen, Denmark; 4 Amsterdam Gastroenterology, Endocrinology, and Metabolism Institute, Amsterdam, the Netherlands; 5 Amsterdam Cardiovascular Sciences Institute, Amsterdam, the Netherlands; 6 Emma Center for Personalized Medicine, Amsterdam UMC, Amsterdam, the Netherlands; 7 Department of Cardiology, Erasmus MC, Rotterdam, the Netherlands; 8 Walter Brendel Centre of Experimental Medicine, University Hospital, Ludwig-Maximilians-Universität Munich, Munich, Germany; 9 Experimental Cardiology, Amsterdam Cardiovascular Sciences, Amsterdam University Medical Centre (UMC), Amsterdam, the Netherlands

**Keywords:** pathophysiology

## Abstract

To better understand pathomechanisms and drug responses in hypertrophic cardiomyopathy (HCM), long-term, reproducible human models for intact cardiac muscle are needed. To this end, we used the MyoDish tissue culture system for a 28-day culture of 73 living myocardial slices (LMSs) derived from myectomy samples of 14 HCM-patients. We defined the model’s applicability in HCM by measuring contractility, myosin super relaxed to disordered relaxed state (SRX-DRX) ratios, mitochondrial function, and metabolic/proteomic profiles in LMS at days (D) 0, 14, and 28. Prominent changes in force, mitochondrial function, protein expression, and metabolism appeared in D14 and D28 slices compared to D0 (non-cultured) slices. Notably, the myosin SRX-DRX ratio and microtubule signatures were retained between D0 and D28, and contractility parameters were unchanged between D8 and D14. Our study indicates that drug interventions targeting these pathways are feasible, though culture conditions require further optimization to fully preserve an HCM-patient-specific profile.

## Highlights

• Long-term cultured HCM myectomy samples show patient heterogeneity as seen clinically
• Living myocardial slices show functional, proteomic and metabolic changes over-time
• Key HCM disease features (relaxation, SRX/DRX, microtubule code) remain unchanged
• Contractility is stable in week 2 and 3 of culture offering a window for drug testing

## Graphical abstract

**Figure:**
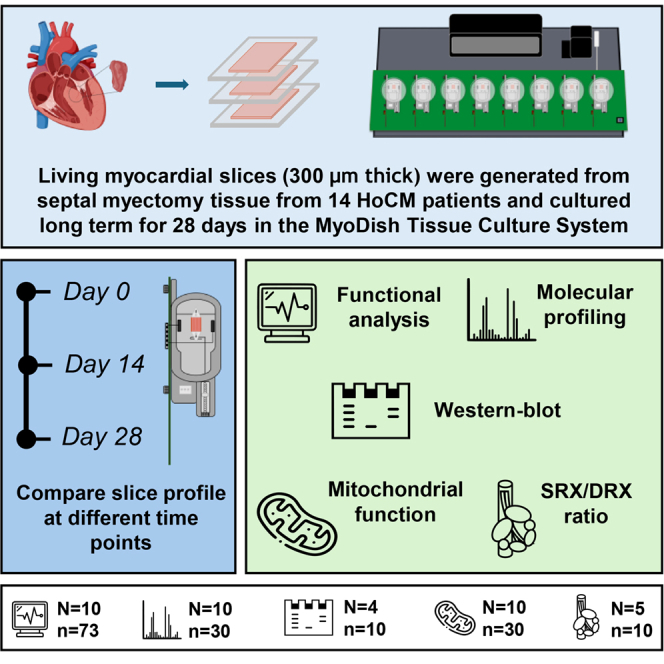

## Introduction

Hypertrophic cardiomyopathy (HCM) is one of the most prevalent forms of cardiomyopathy worldwide^1^ characterized by asymmetric left ventricular (LV) hypertrophy, preserved or slightly elevated systolic function, diastolic dysfunction, cardiomyocyte disarray, fibrosis, and in some cases LV outflow tract obstruction (LVOTO).^2^ Despite the common clinical phenotype, the genetic basis of HCM is diverse and includes pathogenic (P) and likely pathogenic (LP) variants in genes encoding various sarcomere proteins. In approximately 50% of HCM patients, no P/LP gene variant has been identified; these patients are referred to as genotype-negative.^3^^,^^4^^,^^5^^,^^6^ Moreover, the age of symptom onset, progression and severity of disease differ from patient to patient and depend on the presence or absence of a P/LP variant. Thus, a large heterogeneity in HCM exists.

Drug responsiveness may also differ from patient to patient. Current guideline-driven treatment for HCM patients consists of non-invasive therapies to manage arrhythmia, high LV filling pressures and angina, and includes beta-blockers, calcium antagonists and/or disopyramide.^7^ Invasive treatment options include implantable cardioverter-defibrillator placement, and septal reduction therapy to relieve LVOTO by either septal alcohol ablation or septal myectomy surgery. Recently developed drugs for the management of LVOTO are cardiac myosin ATPase inhibitors such as mavacamten and aficamten, which reduce cardiac contractility and workload by normalizing the ratios of myosin super relaxed to disordered relaxed state (SRX-DRX) and are suggested to improve myocardial energetics.^8^^,^^9^ Apart from myosin focused treatments, the microtubules and mitochondria are other potential drug targets to improve cardiac function in HCM patients. Proteomic studies revealed increased detyrosinated α-tubulin in obstructive HCM (HoCM) patients, which directly contributed to diastolic dysfunction.^10^^,^^11^^,^^12^^,^^13^^,^^14^ Hancock et al. showed that the drug colchicine induced microtubule destabilization in healthy rat living myocardial slices (LMSs), improved diastolic performance, along with an increase in myocardial work output.^13^ Similarly, Pietsch et al. provided evidence that activation of tubulin tyrosination improves myocardial function in experimental mouse and human HCM models.^14^ Other studies show that the mitochondria represent a possible target to treat HCM, where *ex vivo* treatment in patient myocardium using NAD^+^ boosters and the drug elamipretide were shown to improve mitochondrial function.^15^ Thus, multiple novel treatments have become available with the potential to prevent, delay and even reverse disease in HCM patients. Notably, different cellular pathomechanisms underlie early and advanced disease stages of HCM. In addition, P/LP gene variant-specific and genotype-specific changes in cardiac dysfunction and remodeling have been reported.^16^^,^^17^ As novel drugs target different cellular mechanisms, they offer the potential for timely patient-specific treatment. LMS represent a potential tool to investigate the acute and chronic effects of drugs, paving the way for an improved understanding of patient-specific drug responsiveness. Metabolic and cytoskeletal remodeling have emerged as key disease pathways in HCM. Targeting these pathways to improve contractile efficiency, relaxation, and hypertrophy will require prolonged treatment. Human LMS can be kept in culture long-term and are therefore useful for testing the chronic effect of drugs. Yet, long-term culture effects on HCM disease characteristics have not yet been determined in human HCM samples. Moreover, using cultured LMS for drug testing requires knowledge about the variation between HCM patient samples. To evaluate the suitability of LMS from HCM patient myocardium as a translational model for chronic drug testing, we first need to determine if HCM disease characteristics are maintained in a long-term culture by tracking the changes that take place over-time and define the most suitable window for drug interventions.

In light of this, we performed contractility analysis by assessing force generation and contractility parameters, Mant-ATP assays to determine myosin’s SRX-DRX ratios, Oroboros experiments to study mitochondrial function, western blot (WB) analysis to quantify protein levels, as well as multi-omics analysis of LMS during a 28-day culture period. Here we report the variation in the properties of LMS from the same patient and among other patient samples, including changes in the functional and metabolic profile of cultured human cardiac tissue. Our study shows that many of the HCM disease characteristics are altered with increasing culture time such as the metabolic and proteomic signature of HCM tissue, while other parameters such as myosin’s SRX-DRX ratios were maintained. All of which should be considered when studying HCM pathomechanisms and testing the effectiveness of acute and chronic drug interventions in long-term cultured human HCM LMS. Taken together, the current model for the *ex vivo* culture of human HCM hearts requires further optimization of culture conditions to recapitulate a complete patient specific profile.

## Results

### Contractility analysis shows a stable window in culture

#### Success rate of long-term culture

Data from ten patient tissue samples were included in the contractility analysis. The MyoDish platform can accommodate up to eight BMCCs/slices. Therefore, eight LMS were prepared from each sample and cultured for 28 days and used for subsequent experiments. In two patients, 350 and 352, the thickness of the tissue sample was less compared to samples received from other patients, and therefore, we could only prepare four and five slices from these samples, respectively (Table 1). On D14 of culture, two patient slices were collected for analysis of mitochondrial function and omics, and the remaining slices were left in culture and were collected after the end of the 28-day culture period for the same analyses. In six out of the ten patients (*n* = 8 slices each), all slices prepared were successfully cultured until being collected on D14 or D28. However, in the other four patients, not all slices continued to beat for 14 or 28 days. From these patients, only 3/8, 4/8, 5/8, and 6/8 prepared slices were successfully cultured beyond the first week.

**Table 1:** Overview of patient data

Patient information						Analysis				
Number	Symbol	Mutation	Sex	Age	Slices					
332		*MYH7* (c.4130C>T) (P)	M	37	8	**✔**	**✔**	–	–	**✔**
348		Not tested	M	62	8	**✔**	**✔**	–	–	**✔**
350		G -	F	67	4	**✔**	**✔**	**✔**	–	**✔**
351		*MYH7* (c.1816G>A) (P)	M	30	8	**✔**	**✔**	**✔**	–	**✔**
352		*CSRP3* (c.131T>C) **(∗)** *TNNT2* (c.832C>T) **(∗)**	F	36	5	**✔**	**✔**	–	**✔**	–
356		G -	F	72	8	**✔**	**✔**	–	**✔**	–
359		G -	M	58	8	**✔**	**✔**	**✔**	**✔**	–
365		G -	M	55	8	**✔**	**✔**	**✔**	**✔**	–
366		Not tested	F	34	8	**✔**	**✔**	–	**✔**	–
368		G -	F	68	8	**✔**	**✔**	–	**✔**	**✔**
370		G -	M	43	8	–	–	–	**✔**	–
371		G -	M	44	8	–	–	–	**✔**	–
372		*MYBPC3* (c.2373dupG) (P)	F	24	8	–	–	–	**✔**	–
373		G -	M	67	8	–	–	–	**✔**	–

Patient information includes a number, symbol, sex, and age (at the time of surgery). If a patient carries a pathogenic (P) variant or a variant with conflicting classifications of pathogenicity **(∗)**, this is indicated. Patients who do not carry a variant are classified as genotype-negative (G-). It is specified the number of slices generated per patient for long term culture, and for which analyses the slices were used.

*CSRP3*, cysteine and glycine rich protein 3; *MYBPC3*, myosin binding protein C3; *MYH7*, myosin heavy chain; *TNNT2*, cardiac troponin T2.

#### Day-to-day variation in force

**Figure 1:**
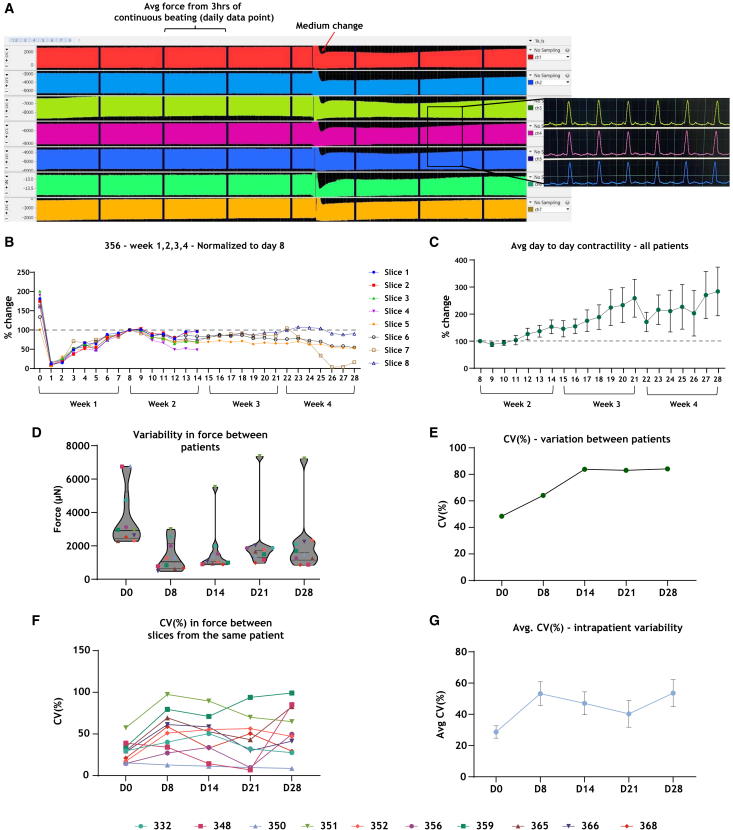
Day-to-day contractility, including inter- and intra-patient variability (A) Example of a 24-h data file divided into 3-h blocks. Data file showing traces of seven slices from the same patient. Zoomed in view showing individual contractions from three slices. (B) Percentage change in force of eight slices from patient 356 over the 28-day culture period normalized to the force of D8. Four slices were used for follow-up experiments on D14. (C) The average day-to-day contractility of ten patients, normalized to the force of D8. (D) Variability between ten patients at different time points in culture, where each data point represents the average force of all slices per patient, per day. ((E) Interpatient variability expressed as the coefficient of variation (CV%) at different time points. (F) Intrapatient variability expressed as the CV% of contractility between the slices from each heart sample at different time points. (G) The average of the CV% of intrapatient variability at different time points in culture. Data in (B and G) are represented as mean ± SEM.

To define the extent of day-to-day variation in force of LMS over 28 days, we used the average force during 3 h of continuous beating for all slices from every patient on every day of culture (Figure 1A). Variability in force among patient slices measured on the same timepoints in culture was found (Figure 1D), and the average coefficient of variance (CV%) on D0, D8, D14, D21, and D28 varied from 45.2%, 61.8%, 82.8%, 82%, to 83%, respectively (Figure 1E). Moreover, the variability in force generation between slices from the same patient (intra-patient variability) was large (Figure 1F), and the average CV% on each time point varied from 28.7% on D0, 53.2% on D8, 47% on D14, 40.3% on D21, to 53.6% on D28 (Figure 1G).

**Figure 2:**
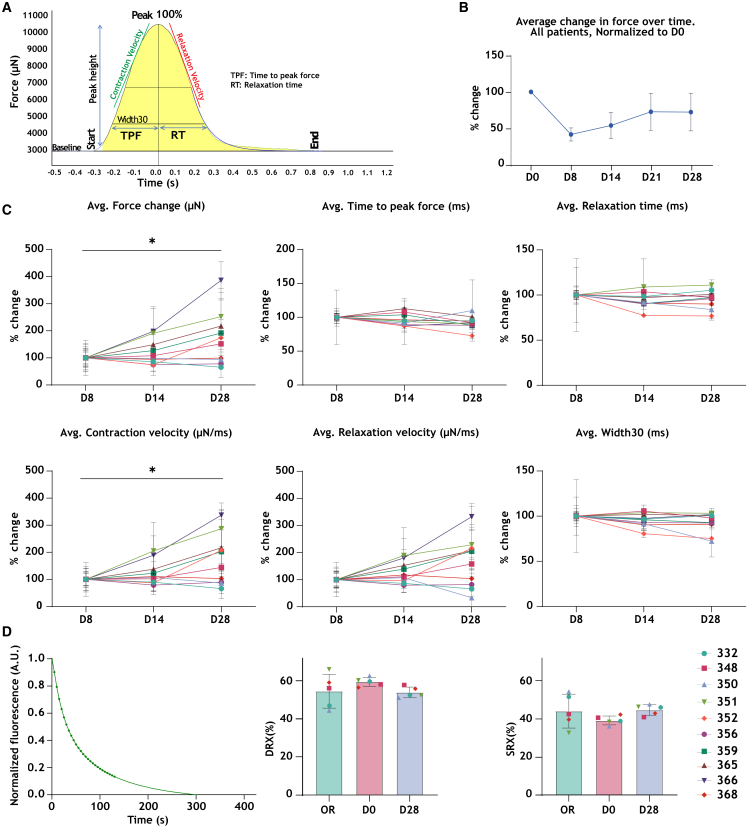
Contractility analysis (A) An illustration of one individual contraction (peak), highlighting the different parameters obtained through the peak analysis module in LabChart Pro 8. (B) The percentage change in force, averaged across all patients at each time point, normalized to D0. (C) Percentage change in contractility parameters over time, normalized to D8. (D) A Mant-ATP assay graph with the average normalized fluorescence of one muscle strip of patient 332. Average percentage DRX and SRX of snap-frozen tissue, non-cultured tissue slices and 28-day cultured slices (five patients). Data in (B–D) are represented as mean ± SEM. Statistical test, one-way ANOVA with Tukey post-hoc test was used. ∗*p* < 0.05.

To assess the stability of force generation during culture, we calculated the average force value from all slices of the same patient on each day and normalized the data to the force value of D0 which was set to 100%, after which an average for each day was calculated for all patients combined (Figure 2B). On D0 of culture, the slices started with generating a strong force of contraction, which significantly declined by D1 to <10% of the initial force (Figure 1B). From D1 onwards, the force of contraction increased day by day until D8 of culture. By D8, force had recovered to 40% of the initial force value of D0 (Figure 2B). During the first week, the slices were completely unstretched and stretched again with 1.5 mN of preload with every medium exchange. This may have contributed to the variability in force observed from day to day during the first week. From D8 onwards, the force of contraction gradually increased during the following 3 weeks of culture; however, only reaching 72% of the initial (D0) force value by D28 (Figure 2B).

#### Contractility parameters

We analyzed different contractility parameters to define changes in force development during the 28-day culture period (Figure 2A). Data were quantified at D8, D14, and D28. When data are normalized to D8, we observed a non-statistically significant increase in force from D8 to D14 (≃ 20%), and from D14 to D28 (≃ 50%) (Figure 2C). Furthermore, the average time to peak force (ms) and relaxation time (ms) did not change over time. The max and min slopes (contraction and relaxation velocities) did not show statistically significant differences at different time points in culture; however, both parameters we increased from D8 to D14 (≃ 23% and 26%, respectively), and from D14 to D28 (≃ 51% and 37%, respectively) (max slope showed a statistically significant difference only when comparing D8 to D28 slices) (Figure 2C). Width 30 showed no differences throughout culture. Taken together, most contractility parameters are stable during culture from day 8–28.

### No change in myosin SRX-DRX ratio during 28 days in culture

A decreased myosin SRX-DRX ratio is observed in HCM patients, and this is the target of the novel myosin ATPase inhibitors. We analyzed whether the time-dependent change in force in LMS is due to alterations in the SRX-DRX state ratio. Mant-ATP assay was performed on tissue samples from five patients. The analysis included OR samples (samples collected and snap-frozen in the operation room), D0 (non-cultured) and D28 LMS. We observed a large heterogeneity in the SRX-DRX state ratio in each patient sample collected at different time points (Figure S1). However, we did not find a statistically significant difference in the averaged SRX-DRX state ratios between samples collected at OR, D0, and D28 (Figure 2D), indicating that the functional state of the myosin heads remains unchanged during a long-term culture of 28 days.

### Mitochondrial function decreases with increasing culture time

**Figure 3:**
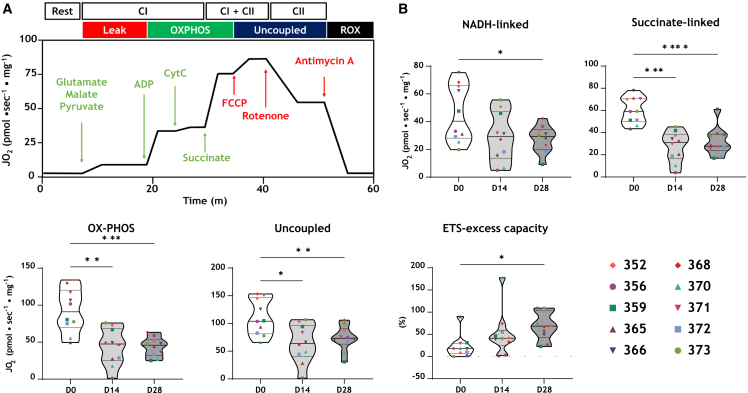
Mitochondrial function (A) Example trace of the mitochondrial respiration experiment. To evaluate mitochondrial respiration, substrates (green) and uncouplers/inhibitors (red) were used. The output trace shows the oxygen consumption. (B) Absolute values of NADH-linked, succinate-linked, OXPHOS and uncoupled respiration were assessed in tissue slices from ten patients at three different time points. The difference between OXPHOS capacity and uncoupled respiration is indicated by the electron transfer system (ETS) excess capacity. Statistical test, one-way ANOVA with Tukey post-hoc test was used. ∗*p* < 0.05, ∗∗*p* < 0.01 and ∗∗∗*p* < 0.001.

We assessed mitochondrial function in fresh LMS at three time points: D0 (non-cultured, immediately after slicing), and on D14 and D28 of culture. The respirometry measurements shown in Figure 3 indicate interpatient variation, which is in accordance with our previous study.^15^ There was a significant decrease in OXPHOS capacity, driven by a reduction in NADH-linked respiration and succinate-linked respiration in cultured slices (D14 and D28) compared to D0 slices (Figure 3B). However, there was no significant difference between D14 and D28 cultured slices. Another parameter that increased with time is ETS (electron transfer system) excess capacity, or spare capacity, with a significant increase observed after 28 days of culture. Generally, all parameters considered indicate that a time-dependent decrease in mitochondrial function took place during the first 2 weeks of culture but remained stable from D14 to D28.

### Omics analysis shows distinct expression profiles between D0 and D14 LMS and similar expression profiles between D14 and D28 LMS

Metabolomics and proteomics were performed on LMS derived from myectomy samples from ten HCM patients to characterize the metabolic and protein expression profile at different time points in culture. We detected 122 metabolites and 1,374 proteins in cultured (D14 and D28) and non-cultured (D0) cardiac slices. The expression profiles of D14 and D28 LMS were compared to the profiles of D0 LMS, and the expression profiles of D14 and D28 LMS were compared to each other. In HCM, altered contractility, ECM remodeling, energy metabolism and disturbed myocardial energetics are some of the main disease-related pathomechanisms.^17^ Therefore, in this report, we mainly highlight changes that occur in these cellular processes. A more in-depth overview of the main changes in the metabolomic and proteomic profiles can be found in the supplementary material S1.

**Figure 4:**
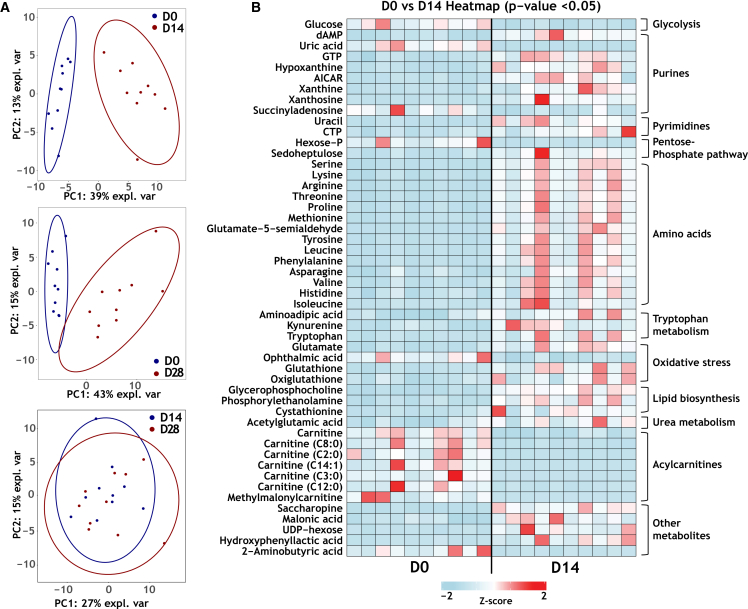
Metabolomics analysis (A) Principal component analysis (PCA) showing the overall expression profile of two groups, with distinct clustering of D0 vs. D14 and D0 vs. D28. (B) Heatmap showing *Z* scores of the top 50 significantly up- or downregulated metabolites between day 0 and day 14. On the left side are all individual metabolites, and on the right is the type of metabolite(s) or the metabolic pathway they belong to.

By performing PCA analysis, we observed that the overall metabolic profile between D0 vs. D14 LMS was distinct (Figure 4A). The same was found when comparing the metabolic profile of D0 vs. D28 LMS (Figure 4A). However, when comparing D14 vs. D28 LMS, we found that samples from these timepoints cluster together, indicating similar abundance patterns of metabolites (Figure 4A). An overview of the top 50 most significant changes in the metabolic profile based on *p* value (<0.05), and Log2 fold-change (>1 and <-1) for D0 vs. D14 can be found in Figure 4B. Significantly changed metabolites between D0 vs. D28 LMS, and between D14 vs. D28 LMS, can be found in Figure S2. The level of glucose in D14 LMS was markedly lower compared to D0 slices. Altered levels of oxidative stress markers were observed, with levels of glutathione and oxiglutathione increasing, and ophthalmic acid decreasing in D14 compared to D0. Furthermore, the level of amino acids was higher, while the level of acylcarnitines was notably lower in D14 compared to D0 slices (Figure 4B). Results from our metabolomics pipeline show that significant changes in metabolite content in LMS take place during the first two weeks of culture and remain stable from D14 to D28.

**Figure 5:**
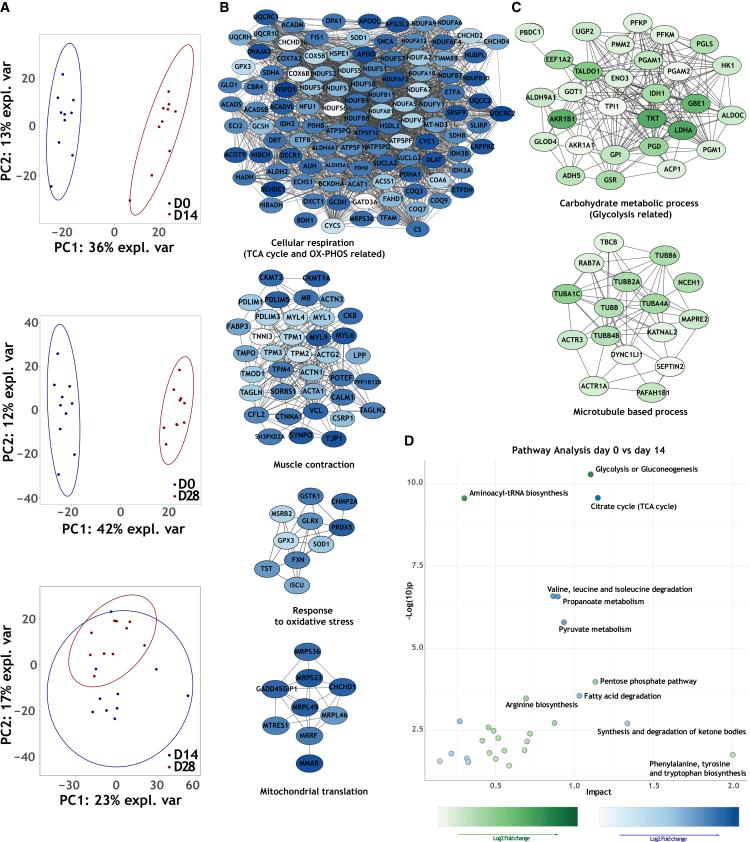
Proteomics analysis (A) Principal component analysis (PCA) showing the overall expression profile of two groups, with distinct clustering of D0 vs. D14 and D0 vs. D28. (B) Down-regulated interacting protein clusters from day 0 to day 14. The different shades of blue indicate the fold-change. (C) Up-regulated interacting protein clusters from day 0 to day 14. The different shades of green indicate fold-change. (D) Metaboanalyst figure showing top up- and down-regulated metabolic pathways based on protein expression.

An overview of the proteomic findings is shown in Figure 5A. PCA analysis showed that D14 and D28 LMS clustered separately from D0, indicating a distinct expression profile upon 14 and 28 days of culture (Figure 5A). ClusterONE and GO analysis were performed using Cytoscape 3.10.2 software, resulting in 41 up-regulated protein pathways, and 37 down-regulated ones in D14 compared to D0 LMS. In D28 LMS, we found 56 up-regulated cellular processes and 39 down-regulated ones compared to D0 LMS. When comparing D28 to D14 LMS, we detected only nine up-regulated and 18 down-regulated cellular processes, and PCA analysis showed similar expression profiles in LMS collected on both time points (Figure 5A). Thus, the proteomics data showed the biggest changes in protein expression were between D0 vs. D14 LMS, and D0 vs. 28 LMS, and the least changes were observed between D14 vs. D28 LMS. Based on relevance to cardiac physiology and the number of proteins involved in every cellular pathway; here, we highlighted the top up- and down-regulated cellular processes. Comparing D0 vs. D14 LMS, cellular respiration and TCA cycle-based metabolism (111 proteins), muscle contraction (37 proteins), Mitochondrion organization (11 proteins), response to oxidative stress (ten proteins), mitochondrial translation (nine proteins), and ECM organization (seven proteins) were downregulated (Figures 5B and S4), while glycolysis (29 proteins) and microtubule-based processes (16 proteins) were upregulated (Figure 5C). Compared to D14 LMS, D28 LMS exhibit similar changes in protein expression profiles when compared to D0 LMS (Figures S5 and S6 – 0 vs. 28). A smaller set of proteins changed from D14 to D28 and included a decreased expression of protein networks regulating oxidative phosphorylation (51 proteins), ventricular action potentials (five proteins), and cardiac contraction (five proteins) (Figures S7 and S8 – 14 vs. 28). Additionally, we constructed Venn diagrams to show a summary of the differentially expressed proteins and overlapping proteins between the different comparisons (D0 vs. D14, D0 vs. D28, and D14 vs. D28) (Figures S11 and S12). The Venn diagrams further confirm that the biggest changes in the protein expression profile take place between D0 vs. D14 LMS, and D0 vs. D28 LMS, while the smallest changes occur between D14 vs. D28 LMS. The data from the proteomics analysis performed indicate that the most changes in protein expression in LMS take place during the first two weeks of culture, and significantly less changes occur between D14 and D28 LMS.

To further understand which cellular pathways are significantly up- or down-regulated in the LMS at different time points, we used the MetaboAnalyst 6.0 online tool for a more comprehensive integration of our metabolomics and proteomics data. The analysis illustrated a significant upregulation of the glycolysis pathway and a downregulation of the TCA cycle pathway in LMS after 14 and 28 days in culture (Figures 5D and S9). When comparing D28 to D14 LMS, there was an upregulation of the pentose phosphate pathway with a fold-change of around 0.7 (Figure S10).

### Decreased expression of sarcomere proteins and the recovery of the microtubule signature in cultured LMS

Since our proteomics data showed an up-regulation of microtubule proteins and a down-regulation of muscle contraction proteins, which may explain changes in contractile parameters during culture, we determined the expression and post-translational modifications (detyrosination, acetylation, and phosphorylation) of specific proteins in D0 (non-cultured), D14, and D28 LMS, as well as snap-frozen tissue (OR). These WB analyses included samples from four patients.

**Figure 6:**
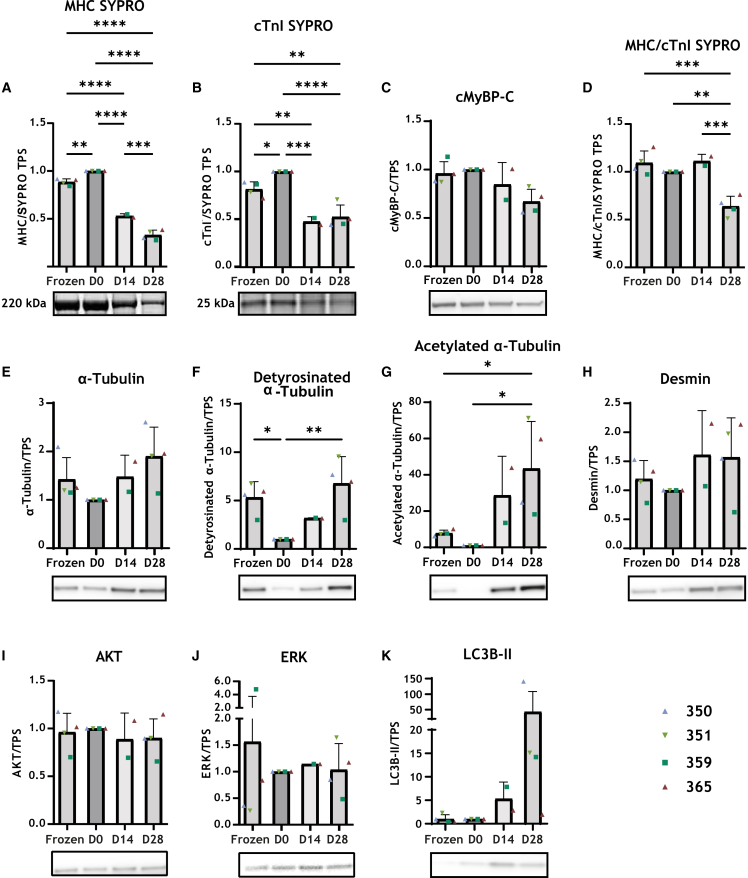
Western blot Staining and quantification of specific cardiac proteins in samples from different time points: frozen tissue at the time of surgery, D0, D14, D28 LMS (A) SYPRO staining of myosin heavy chain (MHC). (B) SYPRO staining cardiac troponin I (cTnI). (C) Cardiac myosin binding protein C (cMYPB-C). (D) Ratio of SYPRO staining for MHC/cTnI. (E) α-tubulin. (F) Detyrosinated α-tubulin. (G) Acetylated α-tubulin. (H) Desmin. (I) AKT. (J) ERK. (K) LC3B-II (autophagy marker). Data are represented as mean ± SD Statistical test, one-way ANOVA with Tukey post-hoc test was used. ∗*p* < 0.05, ∗∗*p* < 0.01, ∗∗∗*p* < 0.001 and ∗∗∗∗*p* < 0.0001.

Microtubule protein expression is altered by a change in temperature, where cold temperatures lead to microtubule depolymerization and a decrease in detyrosination and acetylation.^18^ Accordingly, non-cultured D0 LMS, stored at 4°C after slicing, showed less detyrosinated and acetylated α-tubulin compared to snap-frozen tissue. The levels of microtubule detyrosination and acetylation were restored to the levels of snap-frozen tissue after 14 and 28 days of culture at 37°C. Interestingly, there was no significant change in the level of α-tubulin and desmin proteins between all four time points (Figures 6E–6G).

We also assessed the different levels of cardiomyocyte-specific proteins. In general, phosphorylation levels normalized to total protein levels did not change with time in cultured LMS (data not shown). However, we observed alterations in the level of myosin heavy chain (MHC), cardiac myosin-binding protein C (cMyBP-C), and cardiac troponin-I (cTnI) (Figures 6A–6C), which is in agreement with the proteomic findings. We found an inverse relationship between the protein levels and increasing culture time. This can be an indicator of a decrease in the number of viable cells in LMS throughout culture. In line with this, an increase in the autophagy marker LC3B-II was observed in D14 and D28 LMS, compared to snap-frozen tissue (OR), and D0 LMS, where D28 LMS show the highest expression of this marker (Figure 6K). Furthermore, we then investigated if there are changes in the levels of hypertrophy markers AKT and ERK, to assess whether HCM cardiac slices continue to exhibit hypertrophy in culture and whether the application of 1.5 mN of preload would lead to increased cardiomyocyte hypertrophy during long-term culture. The level of these markers remained unchanged (Figures 6I and 6J). Uncropped WB images can be found in Data S1.

Collectively, our WB experiments confirmed no change in hypertrophy markers, the recovery of the expression of microtubule proteins with increasing culture time and showed time-dependent alterations in sarcomere proteins and LC3B-II.

## Discussion

In this study, we used the MyoDish culture system for a long-term tissue culture using human myectomy tissue samples from HoCM patients who underwent surgery to relieve LVOTO. In this work, we combined contractile and mitochondrial measurements with multi-omics analysis to define the stability of the functional and molecular profile of LMS during a 28-day culture period. We found many long-term culture-related alterations in LMS on days 14 and 28 of culture compared to D0. A sharp but partially reversible reduction in force production was observed during the first week of culture (Figure 1B), while the ratio of SRX-DRX state myosin heads remained unchanged (Figure 2D). After an initial drop, microtubule detyrosination was gradually restored to values observed in snap-frozen tissue at the time of surgery (Figure 6). Notably, prominent metabolic changes were observed on D14 and D28 compared to non-cultured slices on D0 (Figures 3B, 4B, and S2). Cultured LMS became more dependent on glycolytic rather than aerobic metabolism for energy production.

### Transient drop in force

Upon electrical and mechanical stimulation of LMS, the force of contraction was the highest during the first hours of culture on D0 (up to 10 mN). This was followed by a sharp decrease in force from D0 to D1 (≤0.5 mN) (Figure 1B). As discussed elsewhere, this decline in force could be explained by the lack of exposure to physiological stimuli such as hormones and catecholamines.^19^ The force remained low until D8 (less than 50% of D0 force). With time, force gradually increased until the end of the 28 days; however, reaching only 72% of the initial force of D0 (Figures 1B and 2B). Similar findings were reported in other studies that monitored the effect of culture time on force using similar culture conditions,^19^^,^^20^^,^^21^^,^^22^ while a recent study from van der Geest et al., showed that LMS derived from end-stage failing hearts start with a weak force on D0 that gradually increases in a 10-day culture.^23^ Baron et al. demonstrated that hormonal stimulation of LMS was sufficient to preserve contractility over a 6-day culture period in rabbit LMS. Supplementation of culture medium-199 with cortisol and T3 (tri-iodothyronine and thyroid hormone) resulted in maintained levels of contractility on D6 compared to D0 of culture.^22^ Similarly, Miller et al. provided evidence that adding dexamethasone and T3 resulted in preserved cell viability and gene expression in pig LMS after 12 days of culture.^20^ The changes in force in our cultures could not be explained by a change in the conformational states of myosin heads, as the myosin SRX-DRX state ratio in LMS was unchanged throughout the 28 days. This indicated that a long-term culture does not change the HCM phenotype regarding myosin’s functional state and indicates that the chronic effects of myosin-modulating compounds can be studied in cultured human LMS using the current protocol (Figure 2D). Moreover, changes in the level of protein expression did not explain the changes in force with time. A time-dependent decrease in cMyBP-C, cTnI, cTnT, and MHC was observed from D0 to D14 and D28 (Figure 6), while a time-dependent increase in average force was observed in LMS from all patients after day 8 (Figures 1C, 2B, and 2C). In line with the WB analysis, proteomics data also showed a time-dependent decrease in the expression of 37 proteins that regulate muscle contraction from D0 to D14 (Figure 5C), which further decreased from D14 to D28 (Figure S8). This indicates that protein changes at the level of muscle contraction were ongoing till the end of culture, and these changes do not explain the increase in force with time. Moreover, we observed a time-dependent decrease in mitochondrial function (Figure 3) coupled with an increase in the level of detyrosinated and acetylated α-Tubulin (Figures 6F and 6G). Such findings may imply that the force of contraction in LMS is also decreased with time, while the opposite is what we observed. Neither finding could explain why force is partly regained from D8 to D14 to D28. Interestingly, a significant increase was noted in autophagy marker LC3B-II, a key component of cellular protein quality control, (Figure 6K) by D28. The rise in LC3B-II is indicative of an induction of autophagy in LMS, possibly triggered in response to a stressful *ex vivo* culturing environment to remove damaged proteins. Though we did not measure autophagic flux, increased removal of damaged/dysfunctional proteins may underlie the stable force development up to 28 days.^24^

### HCM LMS show large inter- and intra-patient variability for force over-time

Variability in force production among LMS from different patients was vast, though it should be noted that contraction force of LMS from patient 351 had a much higher contraction force on D14, 21, and 28 compared to LMS force values from the other nine patients. Therefore, the high values for CV (%) were driven by one sample (Figure 1E). If the calculation is performed with the exclusion of the data of patient 351, the values for CV (%) on D14, 21, and 28 fall from: 83%, 82%, and 83%, to: 30%, 21%, and 34%, respectively, indicating a much lower variability.

Several factors may contribute to variation between slices. The degree of fibrosis in patient samples may play a role in force variability between slices. Tissues with more fibrosis would have less functional cardiomyocytes and may also influence cardiomyocyte alignment and architecture from patient to patient. Such differences may contribute to the observed variation in force production between patient samples. Similar factors may dictate variations of slice performance in LMS from the same patient, where small differences in slice composition, e.g., the number of cardiomyocytes and their alignment/architecture, could lead to notable differences in force production. Our study shows that there were large variations in contractile performance of LMS from different patients, i.e., the inter-patient variability, while smaller variations in LMS from the same patient sample, i.e., intra-patient variability, were present. Similar findings were presented in LMS from end-stage heart failure (HF) samples cultured for 10 days.^25^ Overall, these data show patient heterogeneity as observed in the clinical setting and a stable patient-specific contractile signature.

### Stable contractility parameters from day 8 until day 14 in culture

Using the current protocol for a long-term culture, our data showed that the first week represents a time frame where LMS undergo the most changes in force generation, while force analysis showed no significant changes in contractility parameters in LMS between D8 and D14 of culture. Accordingly, week 2 is considered the most stable window in a culture of 28 days and could be used for interventions and drug testing to achieve a better interpretation of experimental findings.

### No change in mitochondrial function between D14 and D28 cultured LMS

As new therapies target mitochondria and/or metabolism,^15^^,^^26^ human LMS may represent an excellent model to study the acute and chronic effects of such compounds on mitochondrial and contractile properties. We therefore compared measurements of mitochondrial function in slices on D14 and D28 of culture to non-cultured D0 slices. Oroboros experiments showed a decrease in mitochondrial function during the first 2 weeks of culture, while similar readouts were generated from D14 and D28 LMS (Figure 3B). The level of NADH-linked, succinate-linked, and uncoupled respiration decreased, resulting in a decreased total level of oxidative phosphorylation. The total ETS-excess capacity increased with time, where on D28 the highest average readout was measured (Figure 3B). Excess capacity is defined as the difference between the basal respiratory capacity and the maximum respiratory capacity. Under certain conditions, cells may need a rapid increase in cellular energy to cope with stress or an increased workload. To avoid an “ATP-crisis” inside cardiomyocytes, the reserve capacity has the potential to increase the supply.^27^^,^^28^ Excess capacity is linked to the extent of mitochondrial plasticity, allowing bioenergetic adaptability under stress. An inadequate spare capacity is associated with pathological conditions.^29^ This could explain why lower excess capacity is found in LMS measured directly after slicing on D0, as the pathological phenotype of HCM includes altered bioenergetics and energy demand,^15^ and cardiac slices at that point still require a high demand for ATP. The gradual increase in force observed from D8 to D28 in culture was not coupled with an increase in mitochondrial function. In line with the Oroboros results, metabolomics and proteomics analysis showed a shift in the metabolic profile adapted by LMS, where the slices rely more on glycolytic than aerobic metabolism for energy generation on D14 and D28 compared to D0. Increased levels of TCA cycle substrates such as glutamate, succinate, glutamine, alanine, and pyruvate have been found at D14 vs. D0 (data not shown). Accordingly, we hypothesize that these metabolic substrates were accumulating inside the LMS and were not being used up by the mitochondria. Furthermore, proteomics analysis showed a downregulation of 111 proteins that regulate aerobic metabolism (D0 vs. D14) (Figure 5B), and a further downregulation of 51 of those proteins was found from D14 to D28 (Figure S8). This notion was further confirmed by the metaboanalyst analysis that combined proteomics and metabolomics data. The analysis indicated a significant downregulation of TCA cycle metabolism throughout culture (Figure 5D). A decrease in aerobic respiration was compensated by an increase in glycolytic metabolism. Accordingly, the level of glucose was quickly depleted from LMS (Figure 4B), which could be driven by an increased expression of 29 proteins that regulate glycolysis (Figure 5C). This shift in substrate preference of cardiomyocyte metabolism could be due to the fact that the main fuel for energy metabolism present in the culture medium-199 is glucose (also the addition of ITS-X to the medium). Taken together, this shift in metabolism could be responsible for the decrease in mitochondrial capacity for aerobic respiration shown in D14 and D28 LMS compared to D0 LMS (Figure 3B), and the similarity in metabolic expression profile of D14 compared to D28 LMS could explain the stability in mitochondrial function assessed by Oroboros measurements of slices collected at these two timepoints.

### Downregulation of ECM related proteins with increasing culture time

The ventricular myocardium contains 49.2% ventricular cardiomyocytes, 21.2% mural cells, 15.5% fibroblasts, 7.8% endothelial cells and 5.3% immune cells.^30^ HCM pathology is not only driven by dysfunctional cardiomyocytes but also by their secondary effects on their neighboring cell types and the extracellular matrix (ECM). One of the advantages of using cultured LMS as a disease model is the preservation of the “diseased” ECM at the start of culture. However, in the proteomics data, we showed that a downregulation of a cluster of proteins belonging to the process “ECM organization” was found when comparing D0 to D14 slices. A similar cluster of proteins was also shown to be downregulated when comparing the profile of D0 to D28 LMS. Such changes in ECM components may influence contractile properties of LMS, as the cardiac ECM not only provides structural support to the myocardium, but also acts as a network that allows for force transmission.^31^ As the current culturing conditions do not yet preserve a “diseased” ECM, further adjustments are still needed. In line with our data, similar findings were found when characterizing the changes in cellular processes that occur in cultured LMS of HF patients.^23^

This study provides a comprehensive analysis combining the functional and molecular profile of cultured and non-cultured LMS derived from HoCM patients, providing an integrative view of the remodeling processes during a 28-day culture. Cultured LMS represent a promising model to study cardiac physiology and pathophysiology in more depth, as they recapitulate many of the key *in vivo* cardiac characteristics, including the correct cell ratios and cell types found in the native myocardium, cell-cell communication, and extracellular matrix.^32^ Furthermore, the model introduces a new platform for drug screening and assessing the acute and chronic effects of specific drugs. Here, we showed that key aspects of HCM (e.g., contractile parameters and myosin SRX/DRX ratio) are maintained during a 28-day culture. However, both our study and previous studies that interrogate the integrity of LMS over time^20^^,^^21^ show that further optimization is required in culture conditions to maintain a patient-specific profile without significant remodeling of key disease features in culture. This is considered one of the limitations of using this model in the context of HCM. Based on prior work with pig and rabbit LMS,^20^^,^^21^ we hypothesize that hormonal supplementation of culture medium using cortisol, T3, dexamethasone, or a combination could help preserve the functional and molecular profile of human LMS in long-term cultures.

Notably, myosin, microtubules, and mitochondria are emerging as promising drug targets in HCM. Despite some parameters being unstable in culture, the microtubule signature and myosin conformational states were recovered throughout the culture period. Therefore, exploring treatment options and the study of treatment effects for these drug targets is feasible with the current setup.

### Limitations of the study

Taken together, the findings from this work will provide valuable information for the cardiac research field in HCM by demonstrating the usefulness of the current model, by reporting the changes that occur during a long-term culture of HCM tissue. However, several limitations need to be acknowledged. First, tissue culture experiments were dictated by the size and quality of the obtained septal tissue, and therefore the number of viable LMS produced per patient tissue was not consistent. Second, we only had access to HCM tissue, as no healthy control tissue was available. As a result, it was difficult to determine whether healthy myocardial tissue would respond in the same manner under long-term culture conditions. Finally, all the available sample from the patient tissues were used for the purpose of this work, and therefore no extra samples are available.

## Resource availability

### Lead contact

Requests for further information and resources should be directed to and will be fulfilled by the lead contact, Ali Nassar (a.a.nassar@amsterdamumc.nl).

### Materials availability

This study did not generate new unique reagents.

### Data and code availability

• The proteomics and metabolomics datasets have been deposited at Mendeley data and are publicly available as of the date of publication at https://doi.org/10.17632/mrrxsgfrh2.1.
• This article does not report original code.
• Any additional information required to reanalyze the data reported in this article is available from the lead contact upon request.

## Acknowledgments

J.v.d.V. acknowledges support from 10.13039/501100003246NWO-ZonMW (91818602 VICI grant); the 10.13039/501100001674Leducq Foundation grant number 20CVD01; and the Proper Therapy project funded by the 10.13039/501100003246Dutch Research Council, domain Applied and Engineering Sciences (NWO-AES), the Association of Collaborating Health Foundations (SGF), and ZonMW within the Human models 2.0 call.

## Author contributions

Conceptualization, A.N., V.A.J.W., D.W.D.K., and J.v.d.V.; methodology, A.N., V.A.J.W., D.W.D.K., and J.v.d.V.; investigation, A.N., V.A.J.W., I.D., B.V.S., C.C., and M.v.W.; writing – original draft, A.N., V.A.J.W., I.D., B.V.S., and J.O.; writing – review and editing, A.N., V.A.J.W., I.D., B.V.S., R.H.H., J.O., M.M., A.D., D.W.D.K., and J.v.d.V. ; funding acquisition, J.v.d.V.; resources, M.M. and A.D.; supervision, J.O., R.H.H., A.D., D.W.D.K., and J.v.d.V.

## Declaration of interests

Andreas Dendorfer is the co-founder and shareholder of InVitroSys GmbH, Germany.

Part of this work was funded by a Novo Nordisk Foundation project grant (NNF23-OC0085045) to Julien Ochala.

## STAR★Methods

### Key resources table

**Table:** 

REAGENT or RESOURCE	SOURCE	IDENTIFIER
**Antibodies**		

α-tubulin	Sigma Aldrich T9026	AB_477593
Detyrosinated tubulin	Abcam ab48389	AB_869990
Acetylated tubulin	Sigma Aldrich T7451	AB_609894
Desmin	Cell signaling #5332	AB_1903947
cMyBP-C	Santa Cruz SC-137180	AB_2017317
cTnI	Cell Signaling	4002S
MHC	Produced in-house	N/A
AKT	Cell signaling #9272	AB_329827
ERK	Cell signaling #9102	AB_330744
LC3BII	Cell signaling #2775	AB_915950
ERK	Cell signaling #9102	AB_330744

**Biological samples**		

Left ventricular human septal tissue	HCM patients undergoing septal myectomy surgery	N/A

**Chemicals, peptides, and recombinant proteins**		

d-Glucose	Sigma Aldrich, St. Louis, USA	Cat#:G7021
HEPES	Sigma Aldrich, St. Louis, USA	Cat#: H4034
KCl	Sigma Aldrich, St. Louis, USA	Cat#: P3911
NaCl	Sigma Aldrich, St. Louis, USA	Cat#: S9888
MgCl_2_	Sigma Aldrich, St. Louis, USA	Cat#: M1028
2,3-Butadione Monoxime	Sigma Aldrich, St. Louis, USA	Cat#: B0753
penicillin-streptomycin	Gibco	Cat#: 15140122
Insulin-Transferrin-Selenium-Ethanolamine	Gibco	Cat#: 51500056
Mg-ATP	Sigma Aldrich, St. Louis, USA	Cat#: A9187
Imidazole	Sigma Aldrich, St. Louis, USA	Cat#:I202
EGTA	Sigma Aldrich, St. Louis, USA	Cat#: E4378
K_2_HPO_4_	Sigma Aldrich, St. Louis, USA	Cat#: P3786
Mg acetate	Sigma Aldrich, St. Louis, USA	Cat#: M0631
K acetate	Sigma Aldrich, St. Louis, USA	Cat#: P1190
DTT	Sigma Aldrich, St. Louis, USA	Cat#: D0632
MES hydrate	Sigma Aldrich, St. Louis, USA	Cat#: M8250
2′(3′)-*O*-N-methylanthraniloy-ATP	Invitrogen	Cat#: M12417
KOH	Sigma Aldrich, St. Louis, USA	Cat#: P1767
CaCO_3_	Sigma Aldrich, St. Louis, USA	Cat#: C4830
ATP	Sigma Aldrich, St. Louis, USA	Cat#: A2383
Taurine	Sigma Aldrich, St. Louis, USA	Cat#: T0625
Phosphocreatine	Sigma Aldrich, St. Louis, USA	Cat#: P7936
Sucrose	Sigma Aldrich, St. Louis, USA	Cat#: S8501
fatty acid-free bovine serum albumin	Sigma Aldrich, St. Louis, USA	Cat#: A6003
potassium lactobionate	Sigma Aldrich, St. Louis, USA	Cat#: 153516
Saponin	Sigma Aldrich, St. Louis, USA	Cat#: S7900
Glutamate	Sigma Aldrich, St. Louis, USA	Cat#: G1626
L-malic acid	Sigma Aldrich, St. Louis, USA	Cat#: M1000
sodium pyruvate	Sigma Aldrich, St. Louis, USA	Cat#: P2256
ADP	Merck (Calbiochem)	Cat#: 117105
cytochrome *c*	Sigma Aldrich, St. Louis, USA	Cat#: C7752
Succinate	Sigma Aldrich, St. Louis, USA	Cat#: S2378
carbonyl cyanide-*p*-trifluoro-methoxyphenylhydrazone	Sigma Aldrich, St. Louis, USA	Cat#: C2920
Rotenone	Sigma Aldrich, St. Louis, USA	Cat#: R8875
antimycin A	Sigma Aldrich, St. Louis, USA	Cat#: A8674
Tris-HCl	AppliChem GmbH	Cat#: A1087
Tris base	AppliChem GmbH	Cat#: A1379
LDS	Sigma Aldrich, St. Louis, USA	Cat#: L9781
Glycerol	Sigma Aldrich, St. Louis, USA	Cat#: G7893
EDTA	Invitrogen	Cat#: 15575020
Serva Blue	Serva	Cat#: 35051
Phenol Red	Sigma Aldrich, St. Louis, USA	Cat#: P3532

**Software and algorithms**		

MyoDish Software	InVitroSys https://www.invitrosys.com/	
Metaboanalyst 6.0	www.metaboanalyst.ca	SCR_015539
STRING database	www.string-db.org	SCR_005223
GraphPad Prism 10.0	http://www.graphpad.com/	SCR_002798
Cytoscape	http://cytoscape.org	SCR_003032

### Experimental model and study participant details

#### Ethics committee and approval

Medical Ethics Committee Erasmus University Medical Center. Approval number MEC-2010-40.

#### Study participants

Septal tissue was obtained from 14 HCM patients undergoing septal myectomy surgery. Patient age and sex did not influence any of the methods performed in this manuscript.

#### Patient information

1. Male, 37 years old, Mutation: ***MYH7* (c.4130C>T)**, septal wall thickness: 19 mm
2. Male, 62 years old, Mutation: **not tested**, septal wall thickness: 18 mm
3. Female, 67 years old, Mutation: **G-**, septal wall thickness: 17 mm
4. Male 30 years old, Mutation: ***MYH7* (c.1816G>A)**, septal wall thickness: 21 mm
5. Female 36 years old, Mutation: ***CSRP3* (c.131T>C**) and ***TNNT2* (c.832C>T**), septal wall thickness: 14 mm
6. Female 72 years old, Mutation: **G-,** septal wall thickness: 21 mm
7. Male, 58 years old, Mutation: **G-,** septal wall thickness: 17 mm
8. Male, 55 years old, Mutation: **G-,** septa *l* wall thickness: 17 mm
9. Female, 34 years old, Mutation: **not tested**, septal wall thickness: 14 mm
10. Female, 68 years old, Mutation: **G-,** septal wall thickness: 16 mm
11. Male, 43 years old, Mutation: **G-,** septal wall thickness: 31 mm
12. Male 44 years old, Mutation: **G-,** septal wall thickness: 22 mm
13. Female 24 years old, Mutation: ***MYBPC3* (c.2373dupG)**, septal wall thickness: 18 mm
14. Male, 67 years old, Mutation: **G-,** septal wall thickness: 16 mm

### Method details

#### Human myectomy samples

Interventricular septal tissue was acquired from 14 HoCM patients during septal myectomy surgery to relieve LVOTO. A pathogenic variant was found in 4 of the 14 patients, no variant was found in 8 patients, and 2 patients were not tested. An overview of the patient data can be found in Table 1. All patients signed an informed consent form before their septal tissue was used for this study. The study design and an overview of tissue samples and cardiac slices used for the different analyses at days (D) 0, 14 and 28 are illustrated in the graphical abstract.

#### Cardiac slice preparation and long-term culture

Depending on the degree of the LVOTO in the patient, the obtained myectomy samples varied in size. Myectomy samples were divided into two parts. Part was snap-frozen in liquid nitrogen, and the other part was immediately transferred to ice-cold slicing buffer (4°C) and transported to the laboratory. Slicing buffer consists of Tyrode’s solution (1.0 mM glucose, 5.0 mM HEPES, 5.4 mM KCl, 136 mM NaCl, 1 mM MgCl_2_, 0.9 mM CaCl_2_) and 30 mM 2,3-Butadione Monoxime (BDM), adjusted to pH 7.4 at 4°C. Rapid cooling reduces the energetic demands of the tissue, thereby preventing ischemia and muscle damage.^19^^,^^33^ To enhance the success rate of slicing and long-term culture experiments of LMS, myectomy samples were stored for a maximum of 4–6 h in a cold slicing buffer (4°C) before being used.

##### Tissue handling and slice preparation

Myectomy samples were first examined for regions of poor myofibre alignment or focal fibrosis, which were carefully trimmed away using blunt forceps and a surgical blade without damaging the integrity of the rest of the tissue. The minimum number of acquired slices from one patient tissue sample was 4 slices, and the maximum was 8 slices. Samples were then trimmed into a square or rectangular shape (ideally 0.8–1.0 cm x 0.5–0.8 cm). LMS generated from different patients may have different numbers sizes, and this is solely dependent on the appearance and form of the obtained tissue sample (shape, size, fibrotic tissue composition). The orientation of the myocardial fibers was determined by eye or using light and a magnifying glass if needed. Tissue samples were then embedded in 4% (w/v) low-melting point (LMP) agarose kept at 37°C. The agarose block was left to solidify in a Petri dish on ice for 2–5 min, then mounted on the vibratome (VT1200S, Leica Biosystems, Germany) tray filled with slicing buffer and cooled to 4°C. Vibratome settings were as follows: cutting speed: 0.07–0.10 mm/s, amplitude: 1 mm, slice thickness: 300 μm. Before tissue slicing, a VibroCheck (Leica Biosystems) calibration was performed to ensure precision cutting of the tissue slices with minimal z axis error.

A razor blade was inserted into the vibratome blade holder, and the start/end position of slicing was determined. The blade was oriented perpendicular/tangential to the direction of the myocardial fibers in the sample to ensure smooth cutting of the slices without damage. Embedding the tissue in agarose provides further structural stability to the septal tissue. Automated slicing was then initiated until all the tissue was sliced.

##### Long-term culture

Custom-made plastic triangles were attached to each side of the myocardial fibers using Histoacryl surgical glue (B. Braun, Germany), and the cardiac slices were placed in a biomimetic culture chamber (BMCC) with 2.4 mL of pre-warmed (37°C) culture Medium-199 + 5% penicillin-streptomycin + 1× Insulin-Transferrin-Selenium-Ethanolamine (ITS-X), and 50 μM fresh β-mercaptoethanol (added right before using medium). BMCCs were operated via the MyoDish culture system (InVitroSys, Germany) inside an incubator at 37°C, 5% CO_2_.^19^ The MyoDish was set to 60 rpm rocking speed, and a standard stimulation current of 60 mA (Bipolar pulses, 1 ms charging and discharging pulses separated by 1 ms interval), with a stimulation frequency of 0.5 Hz (30 bpm). Rocking is essential for gas exchange and the equal distribution of medium contents throughout the BMCC. Slices were stretched with 1.5 mN of force to mimic cardiac preload (In the study by Fischer et al.,^19^ it was demonstrated that 1.5 mN of preload is optimal for LMS twitch force in freshly prepared slices). After the first hour of culture, cardiac pre-load was re-adjusted by fully un-stretching the tissue slice and stretching it again with 1.5 mN preload. The force of contraction was constantly recorded and shown on the MyoDish software, and data were stored in 24-h data files (Figure 1A). To access the day-to-day data files from the MyoDish software, data files were first converted to axon binary files (abf) using the MyoDish file converter software, and traces were visualized using the LabChart8 Pro software. The medium was changed every other day by removing 1.6 mL and replacing it with fresh pre-warmed medium. During the first week of culture, the preload was readjusted after each medium exchange. Subsequently, preload was not adjusted anymore, while culture medium was replaced every other day.

#### Contractility analysis

Peak force analysis was performed using the LabChart8Pro software. To monitor day-to-day variation in the force of the LMS from the same patient, data was continuously collected for each cardiac slice during 3 h each day (Figure 1A). Additionally, contractility parameters were analyzed and included: maximal force of contraction (peak height in μN), time to peak force (TPF) (ms), relaxation time (RT) (ms), acceleration to peak (max. slope in μN/s), acceleration to baseline (min. slope in μN/s), and time from 30% of maximal peak force development until 70% to baseline (Width30). TPF was measured from 30% to peak, and RT was measured from peak to 70%. The contractility parameters are illustrated in Figure 2A, and the analysis was performed using the Peak Analysis module in LabChart8 Pro and was based on 30 s of continuous beating (15 contractions). Since the biggest changes in force were observed between D0 and D8, the first stable readout for force was considered after the first week, and therefore, the contractility analysis was normalized to force values of D8, which was set to 100% (Figure 2C).

#### Mant-ATP assay

The ratio of SRX to DRX myosin heads was determined in D0 (non-cultured), D28 LMS, and in snap-frozen tissue at the time of surgery (OR).

As previously published,^34^ the skinning buffer consisted of 50% (v/v) glycerol and 50% (v/v) relaxing solution (4 mM Mg-ATP, 1 mM free Mg^2+^, 10^−6^ mM free Ca^2+^, 10 mM imidazole, 7 mM EGTA, 14.5 mM creatine phosphate). pH and ionic strength were adjusted to 7.0 and 180 mM, respectively. Finally, the rigor buffer, which is essential for running Mant-ATP chase experiments, consisted of 120 mM K acetate, 5 mM Mg acetate, 2.5 mM K_2_HPO_4_, 50 mM MOPS, and 2 mM DTT (pH of 6.8). Five mg of snap-frozen tissue were stored in the skinning solution for 24 h at −20°C and subsequently stored at 4°C for an additional 24 h. Following these procedures, the tissue was kept at −20°C in the same buffer for a maximum of one week before Mant-ATP experiments were carried out.

On the day of testing, thin cardiac strips (1–2 mm long and 50 μm wide) were manually cut in the relaxing buffer. To approximately measure this we used a scale bar under a Petri dish. The construction of our homemade chambers and our specific preparations are described elsewhere.^34^ Briefly, our chambers were placed on a custom-built Zeiss Axio Observer 3 microscope stage, where the sarcomere length of the cardiac strips was assessed using the brightfield mode. For the subsequent measurements, only cardiac strips having a slack sarcomere length of 2 ± 0.05 μm were retained. At 25°C, each cardiac strip within a chamber was initially incubated for 5 min in the rigor buffer. The chamber was then flushed with a rigor buffer having 250 μM 2′(3′)-*O*-(N-methylanthraniloy-ATP (Mant-ATP)). This solution was maintained in the chamber for 5 min. After completing this step, a second rigor solution containing 4 mM unlabelled ATP was introduced, allowing the Mant-ATP chase. For the acquisition, a Plan-Apochromat 20×/0.8 objective and a Zeiss Axiocam 705 mono camera coupled to a Colibry 5 laser were used. Two reference images were acquired just before the Mant-ATP chase. Subsequent images were recorded every 5 s for 5 min (20 ms acquisition/exposure time) using a DAPI filter set, and processed in FIJI 1.54f.^34^ For each cardiac strip, three areas of fluorescent intensity using the batch process function were obtained and normalized to the intensity of the background. Interceptions were set to 1 at *x* = 0 and 0 at *x* = 300*s*. The observed trends were fitted to a two-exponential decay function determined by a nonlinear least square algorithm in GraphPad Prism 10.0.3 Equation model:

$$Normalised\;Flourescence=1-P_{1}\left(1-\exp^{\left(-t/T_{1}\right)}\right)-P_{2}\left(1-\exp^{\left(-t/T_{2}\right)}\right)$$

where P1 is the amplitude of the initial rapid decay (related to DRX) with the time constant T1. P2 is the slower second decay (related to SRX) with its associated time constant T2.

#### Mitochondrial function

Mitochondrial respiration was measured as previously described.^15^ Cardiac slices were measured immediately after slicing (D0, non-cultured) and after D14 and D28 of culture. For direct measurements, slices were transferred from the slicing buffer into a preservation solution (BIOPS solution) containing: 7.2 mM K_2_EGTA, 2.8 mM CaK_2_EGTA, 5.8 mM ATP, 6.6 mM MgCl_2_, 20 mM taurine, 15 mM phosphocreatine, 20 mM imidazole, 0.5 mM dithiothreitol, and 50 mM 2-(N-morpholino) ethane sulfonic acid, with the pH adjusted to 7.1 using KOH. For the cultured LMS, they were taken out of the BMCC, the plastic triangles were removed, and the slices were washed in PBS before being transferred into the BIOPS solution.

All slices were first permeabilized in 2 mL of ice-cold BIOPS solution containing 20 μL of saponin (5 mg/mL) for 30 min on ice while placed on a shaker (90 RPM). Subsequently, the slices were washed twice for 10 min in mitochondrial respiration medium (MIR05) containing: 110 mM sucrose, 1 g/L fatty acid-free bovine serum albumin, 3 mM MgCl_2_, 10 mM KH_2_PO_4_, 20 mM HEPES, 0.5 mM EGTA, 20 mM taurine, 60 mM potassium lactobionate with the pH adjusted to 7.1 using KOH. After washing, the slices were dried, weighed, and transferred into the high-resolution respirometer (Oxygraph-2k; Oroboros Instruments, Innsbruck, Austria). All experimental procedures were performed at 37°C.

To measure leak respiration, 10 mM sodium glutamate, 2 mM sodium malate, and 5 mM sodium pyruvate were added to provide electron transport through complex I. Maximal NADH-linked respiration was then measured after the addition of 5 mM ADP. To assess the integrity of the outer mitochondrial membrane, 10 μM cytochrome *c* was added. Next, to determine OXPHOS capacity, where both complex I and complex II provide electron input, 10 mM succinate was added. The excess capacity of the electron-transferring complexes (I-IV) was assessed by stepwise titration with 0.05 μM FCCP (carbonyl cyanide-*p*-trifluoro-methoxyphenylhydrazone). To evaluate succinate-linked respiration, complex I was blocked by adding 0.5 μM rotenone. Residual oxygen consumption was measured by fully inhibiting mitochondrial oxygen consumption with 2.5 μM antimycin A and was subtracted from all values to obtain the net rates. All measurements were performed in duplicates, and the values were averaged.

#### Multi-omics analysis

Metabolomics and proteomics were performed on snap-frozen LMS from 10 patients at different time points in culture (non-cultured D0, D14, and D28 LMS). Samples from all timepoints were compared to each other to identify long-term culture-related changes in the metabolic and proteomic expression profile of patient-derived LMS. A detailed description of the analytical procedure can be found in the supplemental information. Networks of differentially expressed proteins were analyzed according to the STRING database (www.string-db.org) by uploading raw data files for proteins with *p*-value <0.05 and with Log2-fold change >1 and <-1. Protein networks were then visualized using the Cytoscape 3.10.2 software, where protein clusters were formed using the ClusterONE plug-in, followed by gene ontology analysis (GO) using the BiNGO plug-in. The GO analysis provided insights into which cellular processes the protein networks regulate.

MetaboAnalyst 6.0 (www.metaboanalyst.ca) was used for a comprehensive analysis and integration of the metabolomics with proteomics data, linking both datasets together and showing which processes and pathways were up- and down-regulated at the different time points.

#### Western blot analyses

Snap-frozen tissue was mechanically homogenized with a Dounce homogenizer in reducing sample buffer (RSB) (30 μL/mg). An equal amount of protein (5 μg per sample) was loaded on 4–15% precast Criterion gradient gels (Bio-Rad Laboratories Inc). Western blotting was performed as previously published by Hilderink et al.^12^ Primary antibody concentrations can be found in Table S1. Secondary antibodies (goat anti-rabbit immunoglobulin G-horseradish peroxidase (IgG-HRP, P0448, Dako) and goat anti-mouse IgG-HRP (P0447, Dako)) were incubated at 1:2000. Protein levels were quantified using ImageQuant (Cytiva, USA) and normalized to total protein levels. A more detailed description of the method can be found in the supplemental information.

### Quantification and Statistical analysis

Contractility analysis and Western blot data are presented as the mean ± standard error of the mean (SEM). Oroboros data points represent the average of duplicate measurements. All individual patient data points are color-coded. Statistical analyses were performed using GraphPad Prism v10 software. Data was analyzed with a one-way analysis of variance (ANOVA) followed by Tukey’s multiple comparisons test.

### Additional resources

No additional resources were used.

## Supplemental information

Document S1. Figures S1–S12 and Table S1

Data S1. Uncropped blots
