# Supplementary material for: Ex-vivo culture of human hypertrophic cardiomyopathy hearts: Functional and metabolic changes during long-term culture
Source: iScience. 2026 Mar 11;29(4):115308. doi: 10.1016/j.isci.2026.115308 (PMC13049531; doi:10.1016/j.isci.2026.115308)
Supplement: Document S1. Figures S1–S12 and Table S1 [file mmc1.pdf]

iScience, Volume ■■

## Supplemental information

# *Ex-vivo* culture of human hypertrophic cardiomyopathy hearts: Functional and metabolic changes during long-term culture

**Ali Nassar, Vincent A.J. Warnaar, Inez Duursma, Bauke V. Schomakers, Chahida Chaami, Julien Ochala, Michel van Weeghel, Riekelt H. Houtkooper, Michelle Michels, Andreas Dendorfer, Diederik W.D. Kuster, and Jolanda van der Velden**

# Supplementary material – S1

## METHODS

### *Multi-omics analysis*

Metabolomics, lipidomics and proteomics were performed after a unified extraction method, combining several established methods[S1–S4] . In a 2 mL tube, containing approximately 3 mg of freeze-dried heart tissue, the following amounts of internal standard dissolved in water were added to each sample for metabolomics: adenosine-$^{15}N_5$-monophosphate (5 nmol), adenosine-$^{15}N_5$-triphosphate (5 nmol), $D_4$-alanine (0.5 nmol), $D_7$-arginine (0.5 nmol), $D_3$-aspartic acid (0.5 nmol), $D_3$-carnitine (0.5 nmol), $D_4$-citric acid (0.5 nmol), $^{13}C_1$-citrulline (0.5 nmol), $^{13}C_6$-fructose-1,6-diphosphate (1 nmol), $^{13}C_2$-glycine (5 nmol), guanosine-$^{15}N_5$-monophosphate (5 nmol), guanosine-$^{15}N_5$-triphosphate (5 nmol), $^{13}C_6$-glucose (10 nmol), $^{13}C_6$-glucose-6-phosphate (1 nmol), $D_3$-glutamic acid (0.5 nmol), $D_5$-glutamine (0.5 nmol), $D_5$-glutathione (1 nmol), $^{13}C_6$-isoleucine (0.5 nmol), $D_3$-lactic acid (1 nmol), $D_3$-leucine (0.5 nmol), $D_4$-lysine (0.5 nmol), $D_3$-methionine (0.5 nmol), $D_6$-ornithine (0.5 nmol), $D_5$-phenylalanine (0.5 nmol), $D_7$-proline (0.5 nmol), $^{13}C_3$-pyruvate (0.5 nmol), $D_3$-serine (0.5 nmol), $D_6$-succinic acid (0.5 nmol), $D_4$-thymine (1 nmol), $D_5$-tryptophan (0.5 nmol), $D_4$-tyrosine (0.5 nmol), $D_8$-valine (0.5 nmol)

In the same 2 mL tube, the following amounts of internal standards dissolved in 1:1 (v/v) methanol:chloroform were added for lipidomics: Bis(monoacylglycero)phosphate BMP(14:0)2 (0.2 nmol), Ceramide-1-phosphate C1P (d18:1/12:0) (0.125 nmol), $D_7$-Cholesteryl Ester CE(16:0) (2.5 nmol), Ceramide Cer(d18:1/12:0) (0.125 nmol), Ceramide Cer(d18:1/25:0) (0.125 nmol), Cardiolipin CL(14:0)4 (0.1 nmol), Diacylglycerol DAG(14:0)2 (0.5 nmol), Glucose Ceramide GlcCer(d18:1/12:0) (0.125 nmol), Lactose Ceramide LacCer(d18:1/12:0) (0.125 nmol), Lysophosphatidicacid LPA(14:0) (0.1 nmol), Lysophosphatidylcholine LPC(14:0) (0.5 nmol), Lysophosphatidylethanolamine LPE(14:0) (0.1 nmol), Lysophosphatidylglycerol LPG(14:0) (0.02 nmol), Phosphatidic acid PA(14:0)2 (0.5 nmol), Phosphatidylcholine PC(14:0)2 (2 nmol), Phosphatidylethanolamine PE(14:0)2 (0.5 nmol), Phosphatidylglycerol PG(14:0)2 (0.1 nmol), Phosphatidylinositol PI(8:0)2 (0.5 nmol), Phosphatidylserine PS(14:0)2 (5 nmol), Sphinganine 1-phosphate S1P(d17:0) (0.125 nmol), Sphinganine-1-phosphate S1P(d17:1) (0.125 nmol), Ceramide phosphocholines SM(d18:1/12:0) (2.125 nmol), Sphingosine SPH(d17:0) (0.125 nmol), Sphingosine SPH(d17:1) (0.125 nmol), Triacylglycerol TAG(14:0)2 (0.5 nmol). Subsequently, solvents were added to achieve a total volume of 500 μL water, 500 μL methanol. Muscle tissues were homogenized using a Qiagen TissueLyser II for 2 minutes at 30 times/s with a 5 mm Qiagen Stainless Steel Bead in each tube. Chloroform was added to each tube for a

total chloroform volume of 1 mL, and samples were thoroughly mixed before centrifugation for 10 min at 14.000 rpm to facilitate layer separation.

## Metabolomics

The top layer, containing the polar phase, was transferred to a clean 1.5 mL tube and dried using a vacuum concentrator at 60°C. Dried samples were reconstituted in 100 µL 6:4 (v/v) methanol:water. Metabolites were analyzed using a Waters Acquity ultra-high performance liquid chromatography system coupled to a Bruker Impact II™ Ultra-High Resolution Qq-Time-Of-Flight mass spectrometer. Samples were kept at 12°C during analysis, and 5 µL of each sample was injected. Chromatographic separation was achieved using a Merck Millipore SeQuant ZIC-cHILIC column (PEEK 100 x 2.1 mm, 3 µm particle size). Column temperature was held at 30°C. Mobile phase consisted of (A) 1:9 (v/v) acetonitrile:water and (B) 9:1 (v/v) acetonitrile:water, both containing 5 mmol/L ammonium acetate. Using a flow rate of 0.25 mL/min, the LC gradient consisted of: Dwell at 100% Solvent B, 0-2 min; Ramp to 54% Solvent B at 13.5 min; Ramp to 0% Solvent B at 13.51 min; Dwell at 0% Solvent B, 13.51-19 min; Ramp to 100% B at 19.01 min; Dwell at 100% Solvent B, 19.01-19.5 min. The column was equilibrated by increasing the flow rate to 0.4 mL/min at 100% B for 19.5-21 min. MS data were acquired using negative and positive ionization in full scan mode over the range of m/z 50-1200. Data were analyzed using Bruker TASQ software version 2.1.22.3. All reported metabolite intensities were normalized to freeze-dried tissue weight, as well as to internal standards with comparable retention times and response in the MS. Metabolite identification has been based on a combination of accurate mass, (relative) retention times, ion mobility data and fragmentation spectra, compared to the analysis of a library of standards.

## Lipidomics

The bottom layer, containing the apolar phase, was transferred to a clean 1.5 mL tube and evaporated under a stream of nitrogen at 60°C. The residue was dissolved in 100 µL of 1:1 (v/v) methanol:chloroform. Lipids were analyzed using a Thermo Scientific Ultimate 3000 binary HPLC coupled to a Q Exactive Plus Orbitrap mass spectrometer. For normal phase separation, 2 µL of each sample was injected onto a Phenomenex® LUNA silica, 250 * 2 mm, 5µm 100Å. Column temperature was held at 25°C. Mobile phase consisted of (A) 85:15 (v/v) methanol:water containing 0.0125% formic acid and 3.35 mmol/L ammonia and (B) 97:3 (v/v) chloroform:methanol containing 0.0125% formic acid. Using a flow rate of 0.3 mL/min, the LC gradient consisted of: Dwell at 10% A 0-1 min, ramp to 20% A at 4 min, ramp to 85% A at 12 min, ramp to 100% A at 12.1 min, dwell at 100% A 12.1-14 min, ramp to 10% A at 14.1 min, dwell at 10% A for 14.1-15 min. For reversed phase separation, 5 µL of each sample was injected onto a Waters

HSS T3 column (150 x 2.1 mm, 1.8 µm particle size). Column temperature was held at 60°C. Mobile phase consisted of (A) 4:6 (v/v) methanol:water and B 1:9 (v/v) methanol:isopropanol, both containing 0.1% formic acid and 10 mmol/L ammonia. Using a flow rate of 0.4 mL/min, the LC gradient consisted of: Dwell at 100% A at 0 min, ramp to 80% A at 1 min, ramp to 0% A at 16 min, dwell at 0% A for 16-20 min, ramp to 100% A at 20.1 min, dwell at 100% A for 20.1-21 min. MS data were acquired using negative and positive ionization using continuous scanning over the range of m/z 150 to m/z 2000. Data were analyzed using an in-house developed lipidomics pipeline written in the R programming language (http://www.r-project.org). All reported lipids were normalized to corresponding internal standards according to lipid class, as well as to freeze-dried tissue weight. Lipid identification has been based on a combination of accurate mass, (relative) retention times, fragmentation spectra, analysis of samples with known metabolic defects, and the injection of relevant standards.

NB: Lipidomics was performed, but the data was not used for this study.

### Proteomics

After the transfer of both solvent layers, the remaining protein pellet was dried under a stream of nitrogen. Proteomics sample preparation was performed using a Thermo Scientific™ EasyPep™ MS Sample Prep Kit (A40006), according to the kit's instructions. Briefly, 200 µL lysis buffer was added to each sample, and a Thermo Scientific™ Pierce™ BCA Protein Assay (23225) was performed according to kit instructions to determine protein content. For each sample, 100 µg of protein was transferred to a clean 2 mL tube. Samples were reduced and alkylated at 95°C for ten minutes, followed by a two-hour incubation at 37°C with a Trypsin/Lys-C protease mixture. After sample clean-up with the Peptide Clean-up Plate, samples were dried under a stream of nitrogen at 60°C, before being resuspended in a 100 µL mixture of 97:3 (v/v) water:acetonitrile, containing 0.1% formic acid.

Samples were kept at 12°C during analysis, and 10 µl of each sample was injected. Injection order for samples was random, with injections of a pooled sample at the start and end, as well as at varying intervals throughout the series. Chromatographic separation was achieved on a Waters™ Acquity UPLC, using a Waters™ Acquity UPLC BEH C18 Column (130Å, 1.7 µm, 2.1 mm X 50 mm)(186002350), equipped with a Waters™ Acquity UPLC Vanguard BEH C18 precolumn (186003975). Column temperature was held at 60°C. The mobile phase consisted of (A) water and (B) acetonitrile, both containing 0.1% formic acid. Using a starting flow rate of 0.5 ml/min, the LC gradient consisted of: Dwell at 3% B for 0-0.1 min; Ramp to 40% B at 4.3 min; Ramp to 80% B at 4.31 min with a flow rate of 0.85 ml/min; Dwell at 80% B for 4.31-4.40 min with a flow rate of 0.85 ml/min; Ramp to 3% B at 4.50 min with a flow rate of 0.6 ml/min; Dwell at 3% B for 4.5-5 min with a flow rate of 0.5 ml/min.

MS data were acquired with a Bruker timsTOF Pro 2 using positive ionization in DIA-PASEF mode as previously reported[S3] .

DIA-PASEF data files were processed using DIA-NN version 1.8.1[S5], using a spectral library provided by Bruker. Data were normalized in DIA-NN using MaxLFQ [S6].

## *Western Blot*

Flash frozen tissue slices have been mechanically homogenized with a dounce in reducing sample buffer (RSB)(30µl/mg). An equal amount of protein per sample was loaded on 4-15% precast Criterion™ gradient gels (Bio-Rad Laboratories Inc) and ran in sodium dodecyl sulfate (SDS) electrophoresis buffer for 90 min at 100V. Wet tank membrane transfer onto PVDF membranes ran for 120min at 0.3A, after which total protein stains were performed and imaged using the Odyssey XF (LI-COR Biosciences, USA). The membranes were blocked in 5% (w/v) milk or 3% (w/v) bovine serum albumin (BSA) in tris-buffered saline with 0.1% (v/v) tween (TBS-T). Primary antibodies were incubated in 3% BSA in TBS-T overnight at 4°C. After washing the membranes in TBS-T, secondary antibodies (goat anti-rabbit immunoglobulin G-horseradish peroxidase (IgG-HRP, P0448, Dako) and goat anti-mouse IgG-HRP (P0447, Dako)) were incubated in 3% BSA in TBS-T at 1:2000 for 1h at room temperature (RT). Membranes were washed, incubated with equal amounts of solution A and B of the Amersham™ ECL Select™ Western Blotting Detection Reagent kit and imaged using the Amersham Imager 600 (GE Healthcare Bio-Sciences AB). Antibody concentrations can be found in Table 1 . Protein levels were quantified using ImageQuant (Cytiva, USA) and normalized to total protein levels.

| **Target** | **Company/product number** | **Dilution** |
|---|---|---|
| α-tubulin | Sigma Aldrich T9026 | 1:2000 |
| Detyrosinated tubulin | Abcam ab48389 | 1:1000 |
| Acetylated tubulin | Sigma Aldrich T7451 | 1:10000 |
| Desmin | Cell signaling #5332 | 1:1000 |
| cMyBP-C | Santa Cruz SC-137180 | 1:5000 |
| cTnI | Cell Signaling #4002S | 1:2500 |
| cTnT | Abcam ab10214 | 1:1000 |
| MHC | Produced in-house | 1:5000 |
| AKT | Cell signaling #9272 | 1:1000 |

| | | |
|---|---|---|
| ERK | Cell signaling #9102 | 1:1000 |
| LC3BII | Cell signaling #2775 | 1:1000 |

**Table S1.** List of antibodies used and their concentrations.

# RESULTS

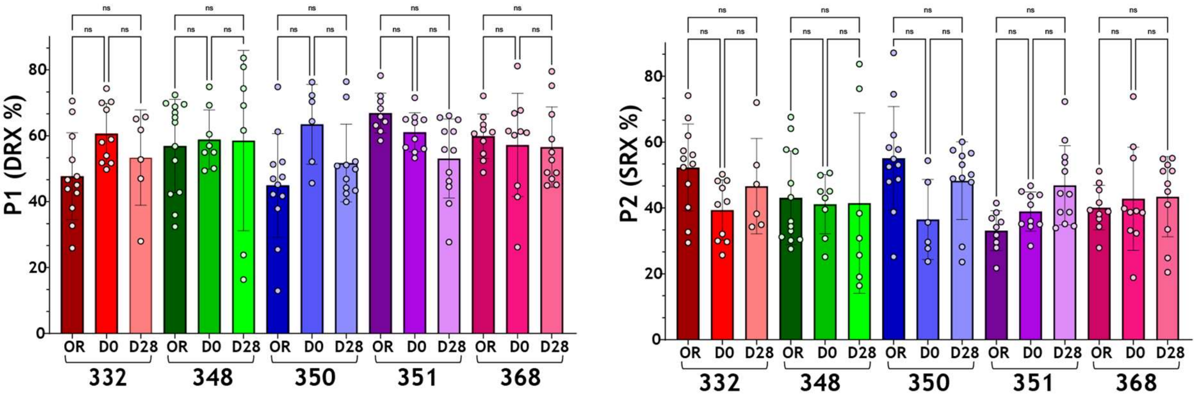


**Figure S1. Heterogeneity in Mant-ATP assay**.

Ratio of myosin SRX:DRX in LMS from 5 patient samples. Experiments were performed on snap-frozen tissue (OR), D0 and D28 LMS.

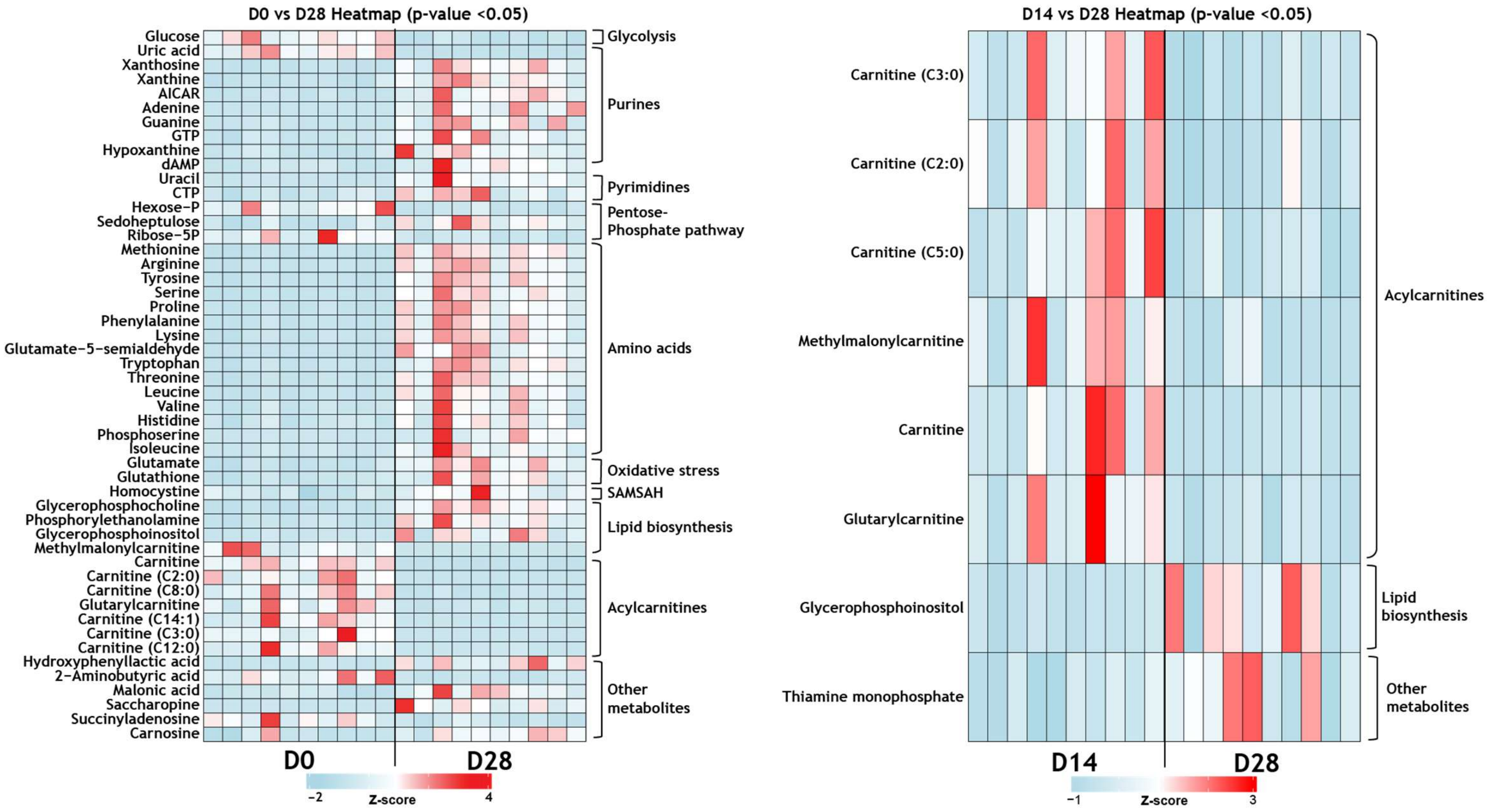


**Figure S2. Metabolomics data.**

Heatmaps showing z-scores of the top significantly up- or downregulated metabolites between D0 vs D28 LMS (left), and D14 vs D28 LMS (right). On the left side of the heatmap are all individual metabolites, and on the right are the metabolic pathways they belong to.

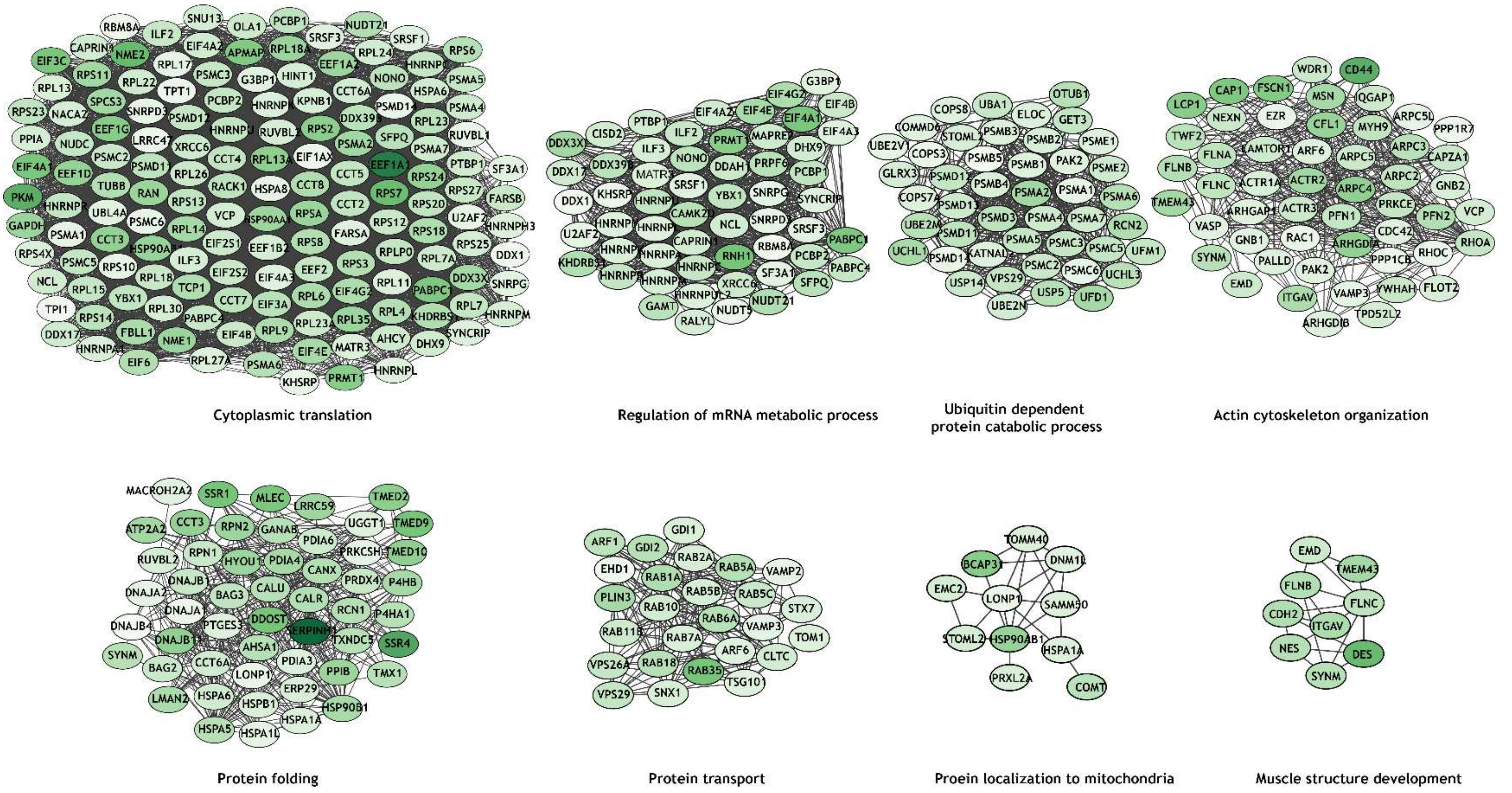


**Figure S3. Proteomics Analysis**.

Top upregulated protein networks in D0 vs D14 LMS.

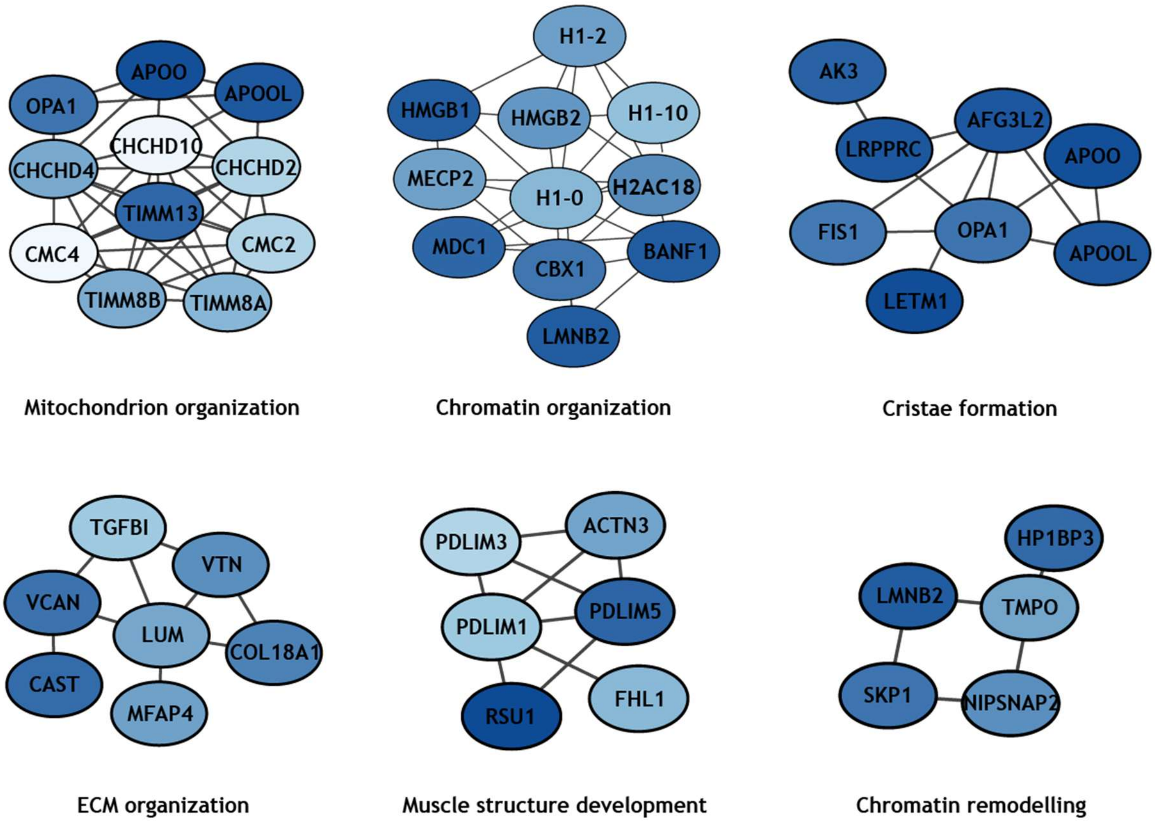


**Figure S4. Proteomics analysis**.

Top downregulated protein networks in D0 vs D14 LMS.

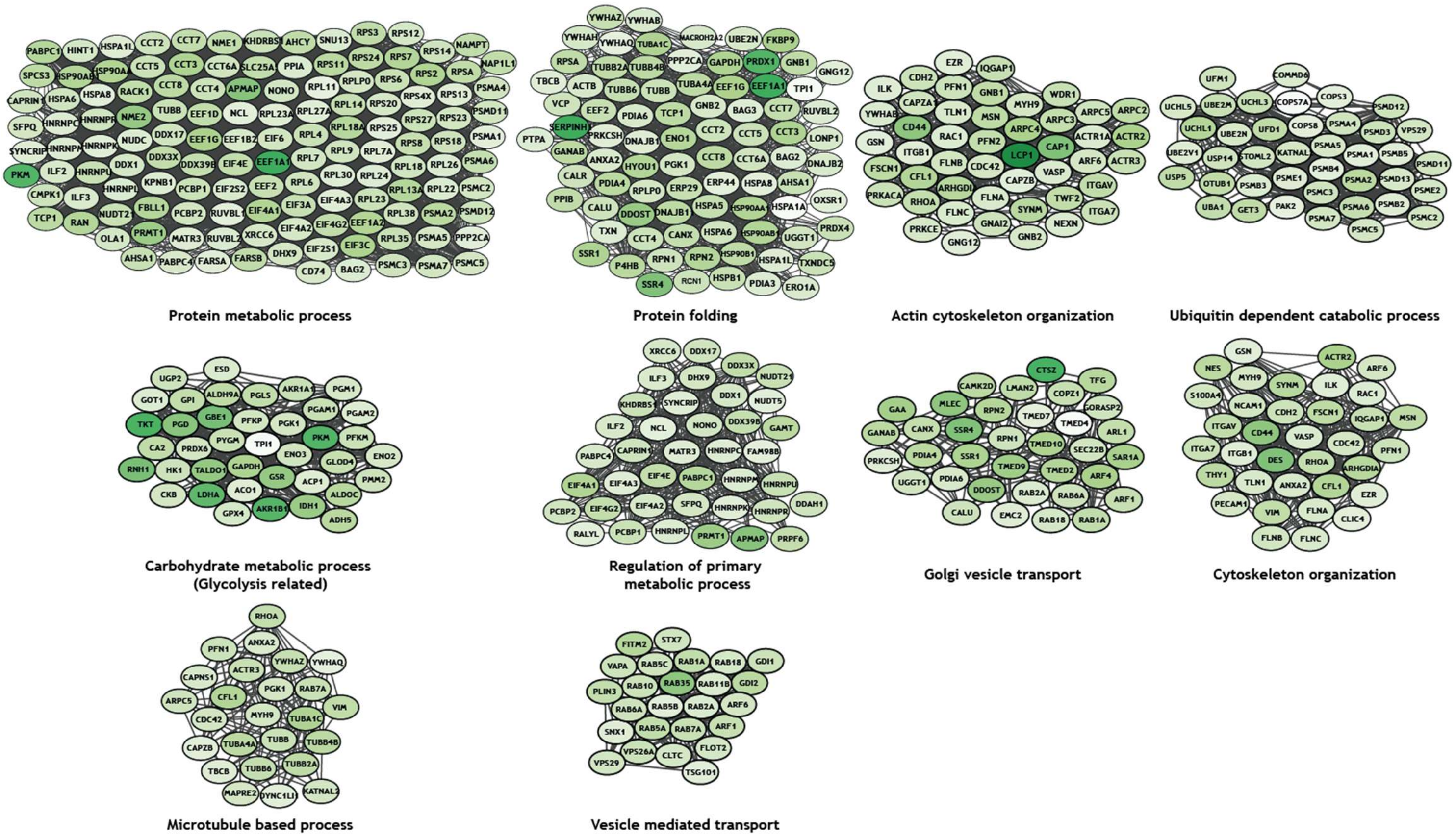


**Figure S5. Proteomics analysis.**

Top upregulated protein networks in D0 vs D28 LMS.

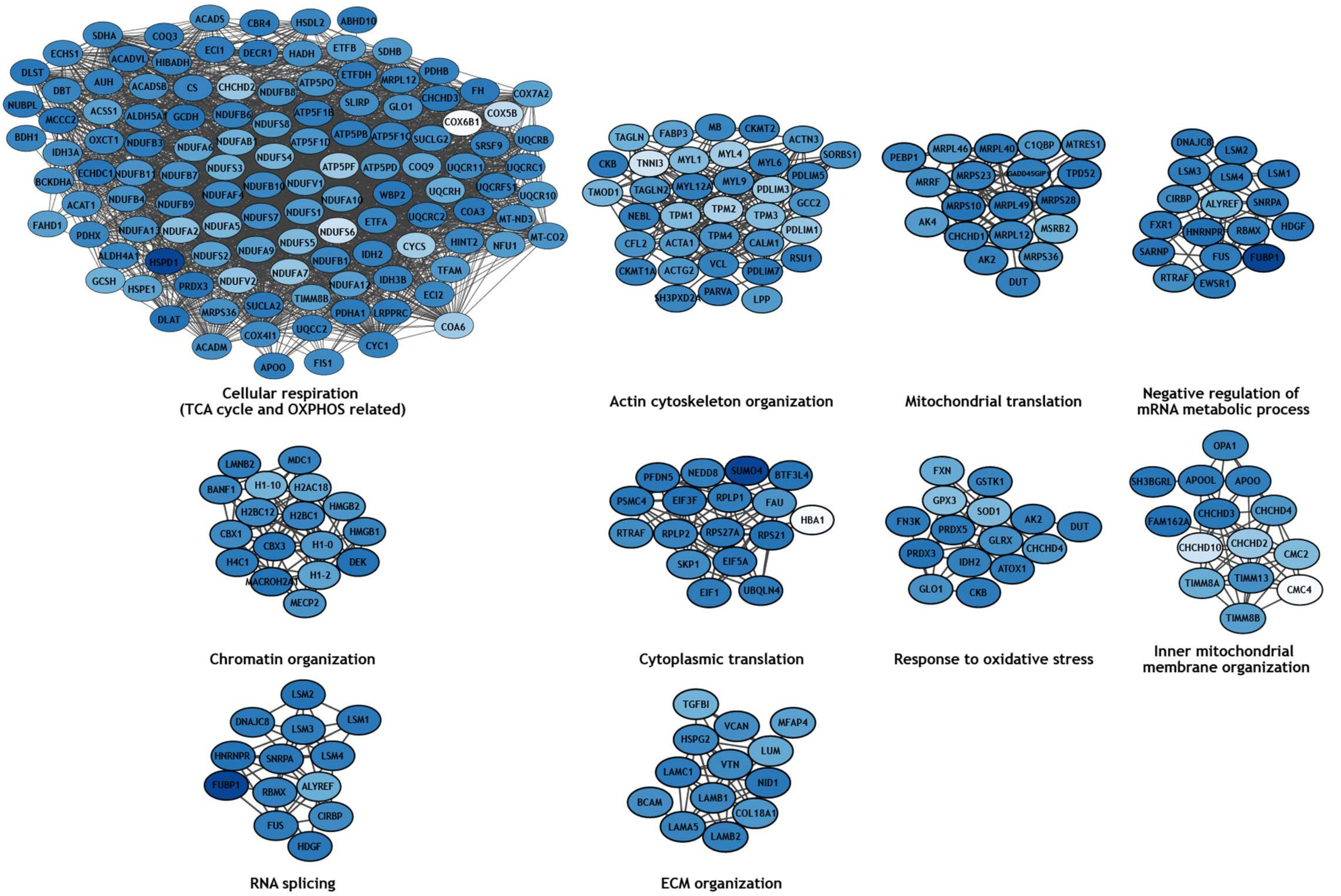


**Figure S6. Proteomics analysis.**

Top downregulated protein networks in D0 vs D28 LMS.

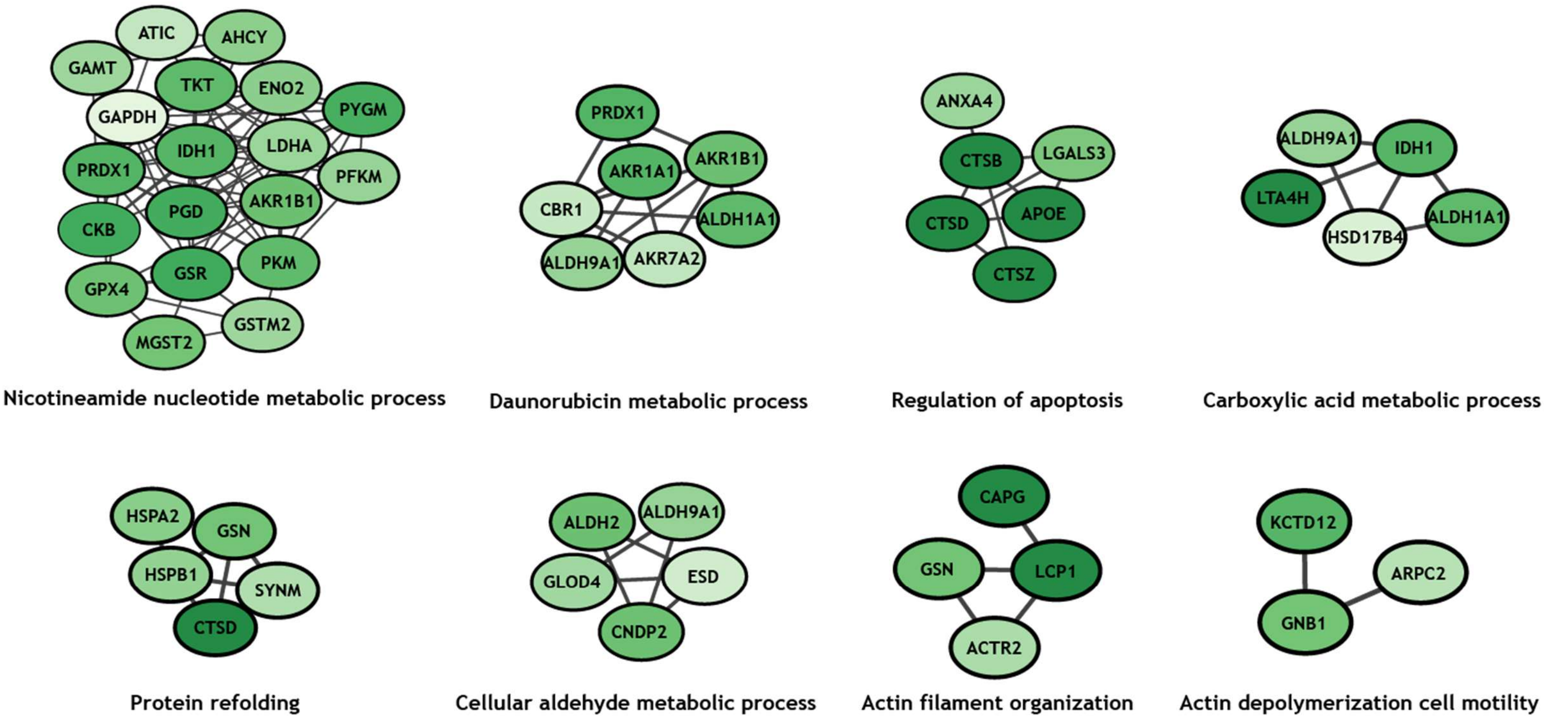


**Figure S7. Proteomics analysis.**

Top upregulated protein networks in D14 vs D28 LMS.

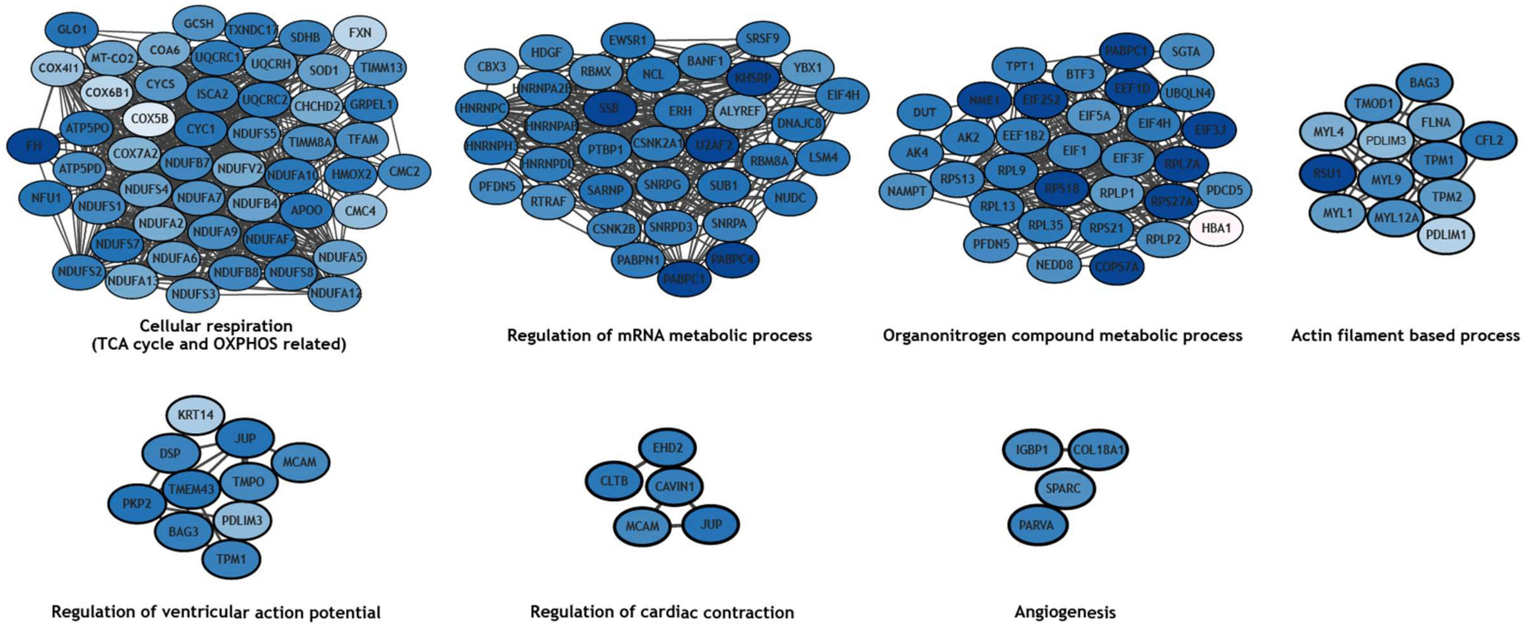


**Figure S8. Proteomics analysis.**

Top downregulated protein networks in D14 vs D28 LMS.

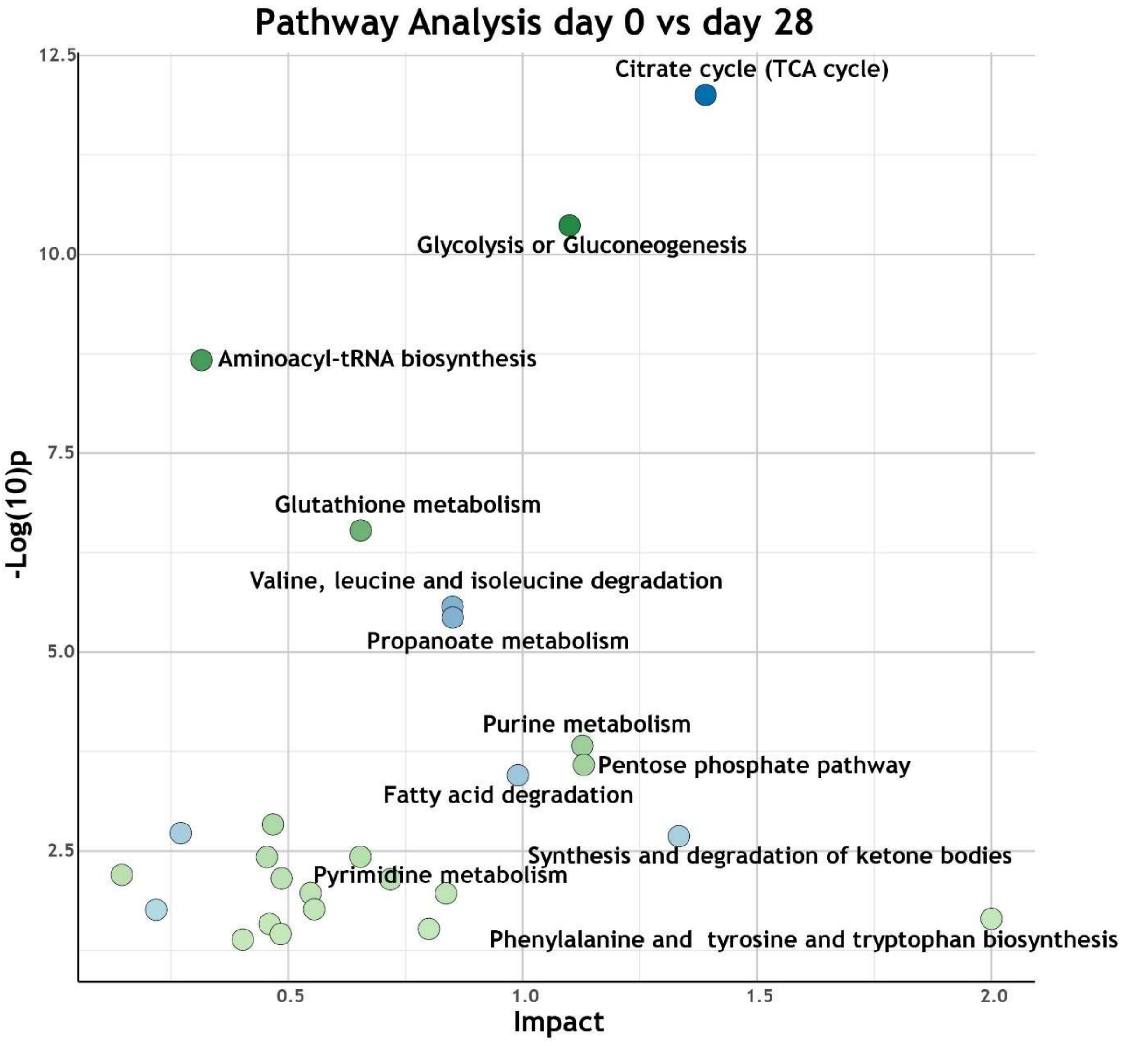


**Figure S9. Combining metabolomics and proteomics data.**

Metaboanalyst pathway analysis comparing D0 vs D28 LMS and showing top up- and down-regulated cellular pathways. Green: upregulated, Blue: downregulated.

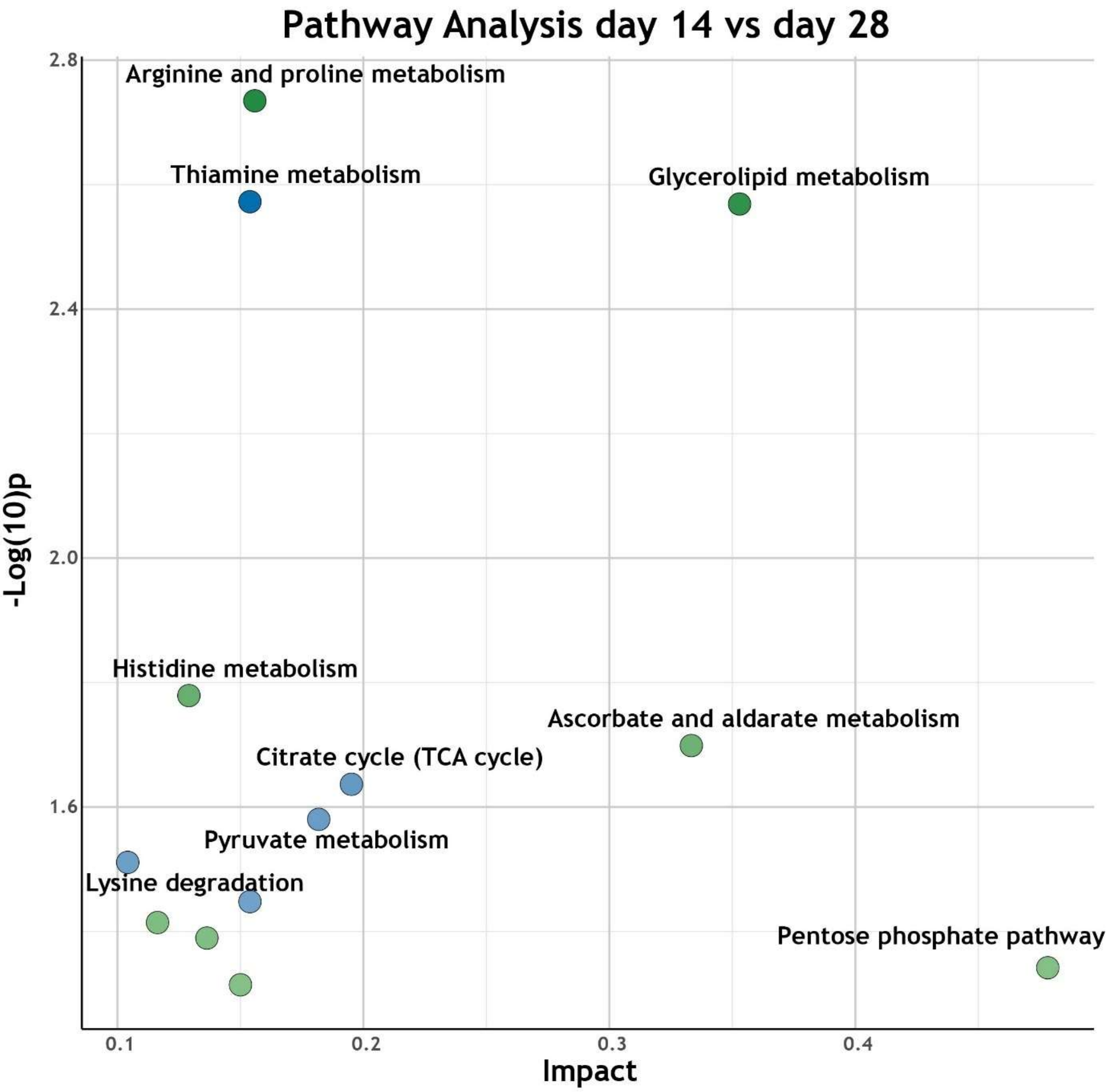


**Figure S10. Combining metabolomics and proteomics data.**

Metaboanalyst pathway analysis comparing D14 vs D28 LMS and showing top up- and down-regulated cellular pathways. Green: upregulated, Blue: downregulated.

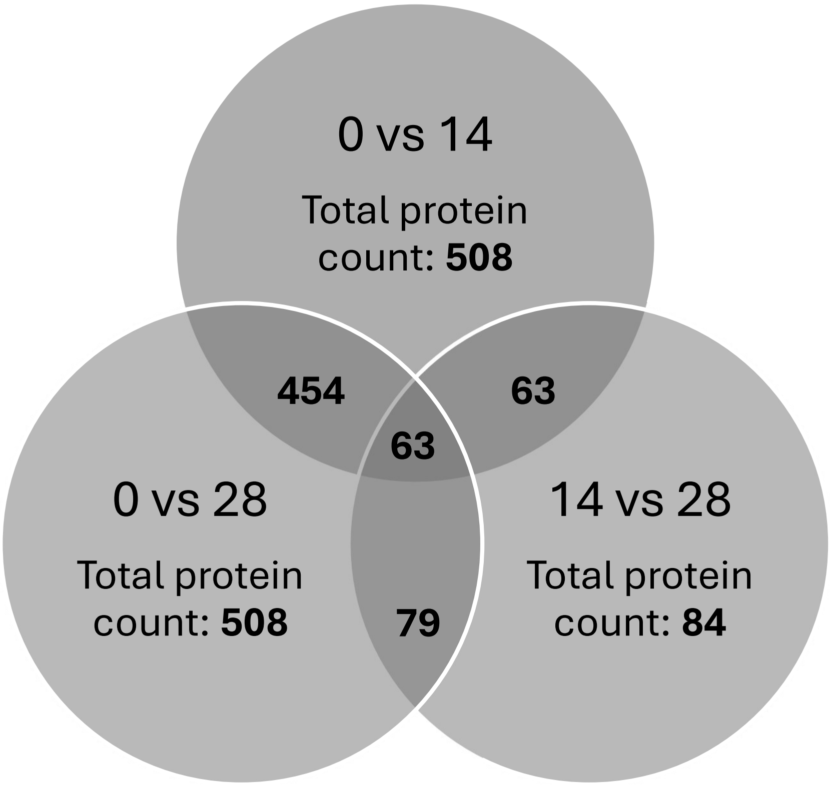


**Figure S11. Summary of upregulated proteins in LMS.**

Overlapping proteins between the comparison of D0 vs D14, D0 vs 28, and D14 vs D28 LMS.

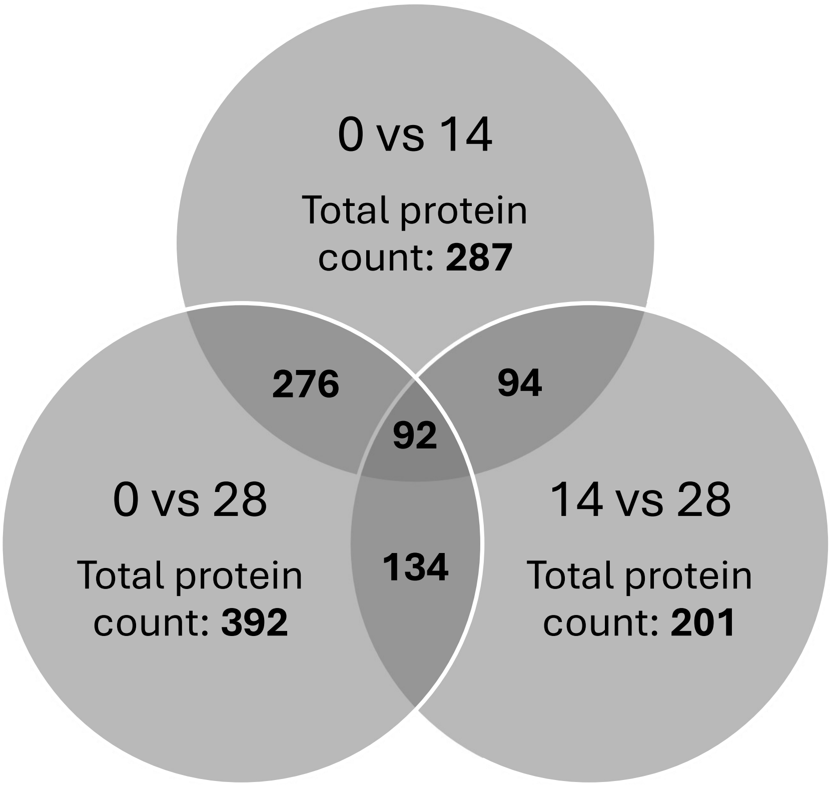


**Figure S12. Summary of downregulated proteins in LMS.**

Overlapping proteins between the comparison of D0 vs D14, D0 vs 28, and D14 vs D28 LMS.

## REFERENCES


S1. Schomakers, B. V. *et al.* Polar metabolomics in human muscle biopsies using a liquid-liquid extraction and full-scan LC-MS. *STAR Protoc* **3**, (2022). DOI: 10.1016/j.xpro.2022.101302 .

S2. Molenaars, M. *et al.* Metabolomics and lipidomics in Caenorhabditis elegans using a single-sample preparation. *DMM Disease Models and Mechanisms* **14**, (2021). DOI: 10.1242/dmm.047746.

S3. Szyrwiel, L., Gille, C., Mülleder, M., Demichev, V. & Ralser, M. Fast proteomics with dia-PASEF and analytical flow-rate chromatography. *Proteomics* **24**, 1–9 (2024). DOI: 10.1002/pmic.202300100.

S4. Schomakers, B. V *et al.* Integrated Multi-Omics Mapping of Mitochondrial Dysfunction and Substrate Preference in Barth Syndrome Cardiac Tissue. (2025). DOI: 10.1038/s44321-025-00320-5

S5. Demichev, V., Messner, C. B., Vernardis, S. I., Lilley, K. S. & Ralser, M. DIA-NN: neural networks and interference correction enable deep proteome coverage in high throughput. *Nat Methods* **17**, 41–44 (2020). DOI: 10.1038/s41592-019-0638

S6. Cox, J. *et al.* Accurate proteome-wide label-free quantification by delayed normalization and maximal peptide ratio extraction, termed MaxLFQ. *Molecular and Cellular Proteomics* **13**, 2513–2526 (2014). DOI: 10.1074/mcp.M113.031591
