# Supplementary material for: Ex-vivo culture of human hypertrophic cardiomyopathy hearts: Functional and metabolic changes during long-term culture
Source: iScience. 2026 Mar 11;29(4):115308. doi: 10.1016/j.isci.2026.115308 (PMC13049531; doi:10.1016/j.isci.2026.115308)
Supplement: Data S1. Uncropped blots [file mmc2.pdf]

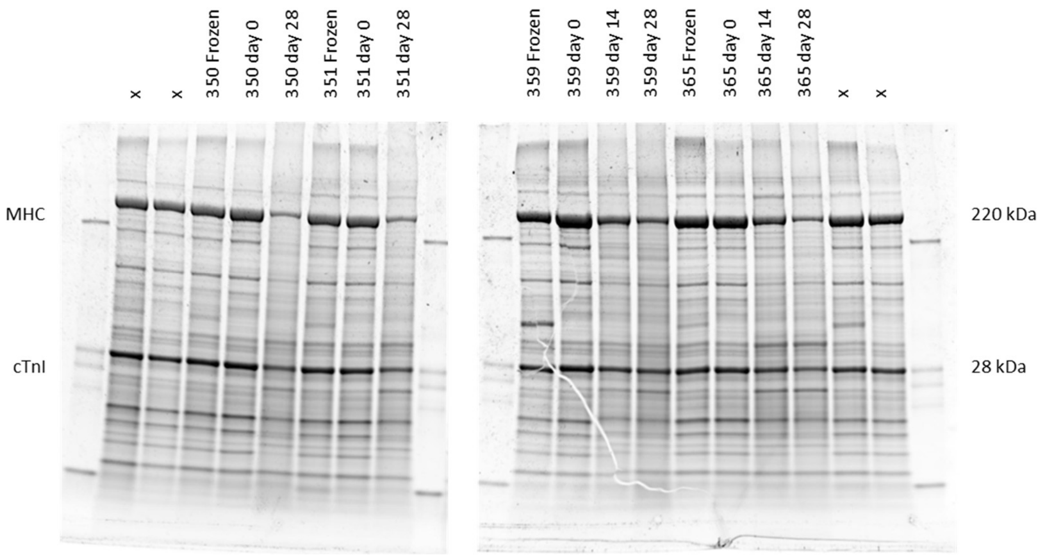


Uncropped images of a SYPRO staining corresponding to the MHC and cTnI quantification in figure 6. Membranes were cut for imaging purposes only. Membranes were imaged using the Amersham Imager 600 (GE Healthcare Bio-Sciences AB). Protein expression was normalized against the total protein in the SYPRO staining. X = samples that were not included in the analysis.

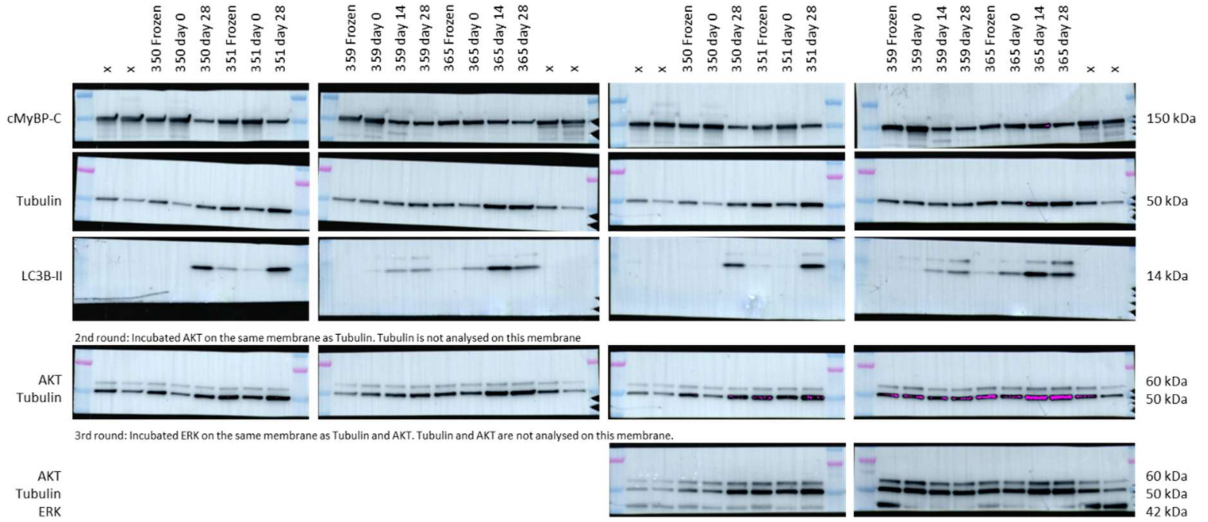


Uncropped western blots of run1 corresponding to figure 6. Membranes were cut for imaging purposes only. Membranes were incubated with enhanced chemiluminescent (ECL) detection reagent (Amersham) and imaged using the Amersham Imager 600 (GE Healthcare Bio-Sciences AB). Protein expression was normalized against the total protein stain. X = samples that were not included in the analysis.

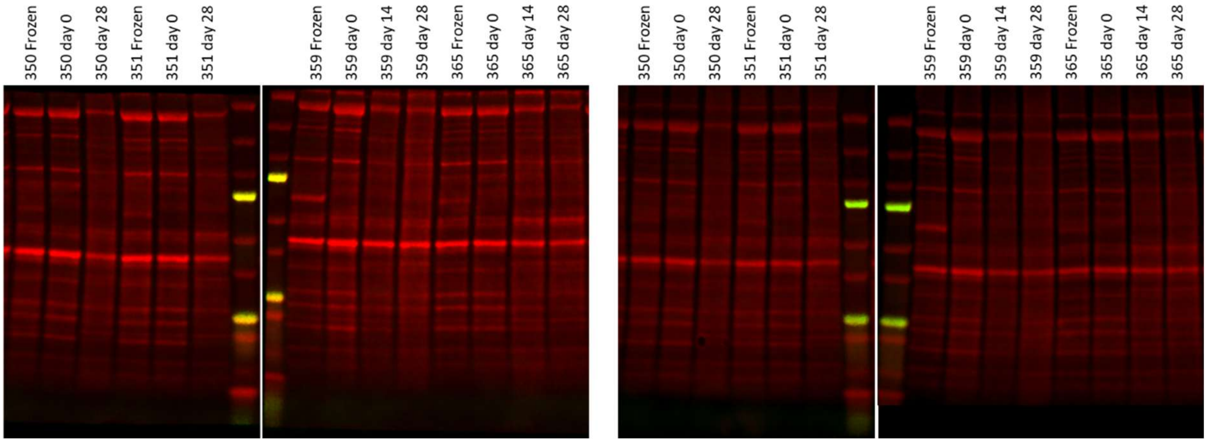


Total protein stain belonging to run 1.

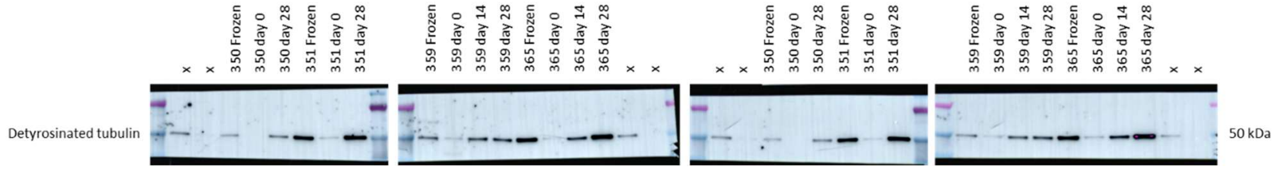


Uncropped western blots of run 2 corresponding to figure 6. Membranes were cut for imaging purposes only. Membranes were incubated with enhanced chemiluminescent (ECL) detection reagent (Amersham) and imaged using the Amersham Imager 600 (GE Healthcare Bio-Sciences AB). Protein expression was normalized against the total protein stain. X = samples that were not included in the analysis.

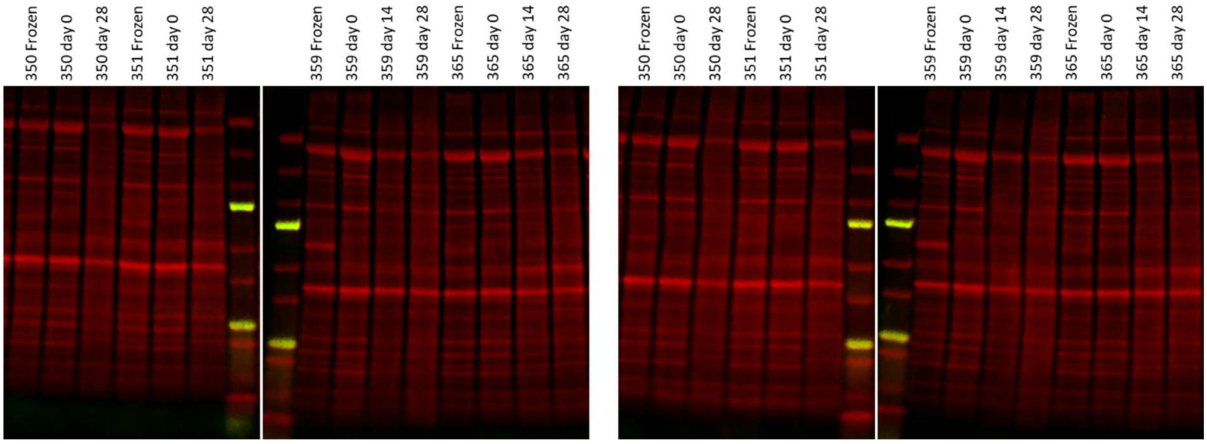


Total protein stain belonging to run 2.

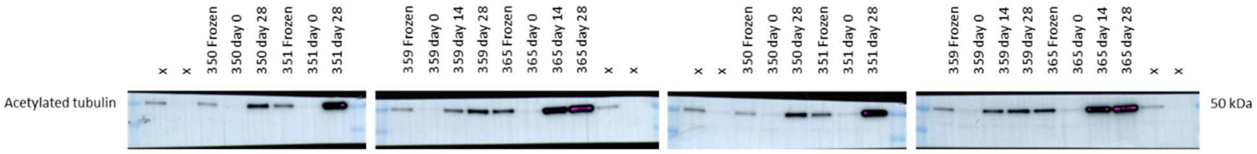


Uncropped western blots of run 3 corresponding to figure 6. Membranes were cut for imaging purposes only. Membranes were incubated with enhanced chemiluminescent (ECL) detection reagent (Amersham) and imaged using the Amersham Imager 600 (GE Healthcare Bio-Sciences AB). Protein expression was normalized against the total protein stain. X = samples that were not included in the analysis.

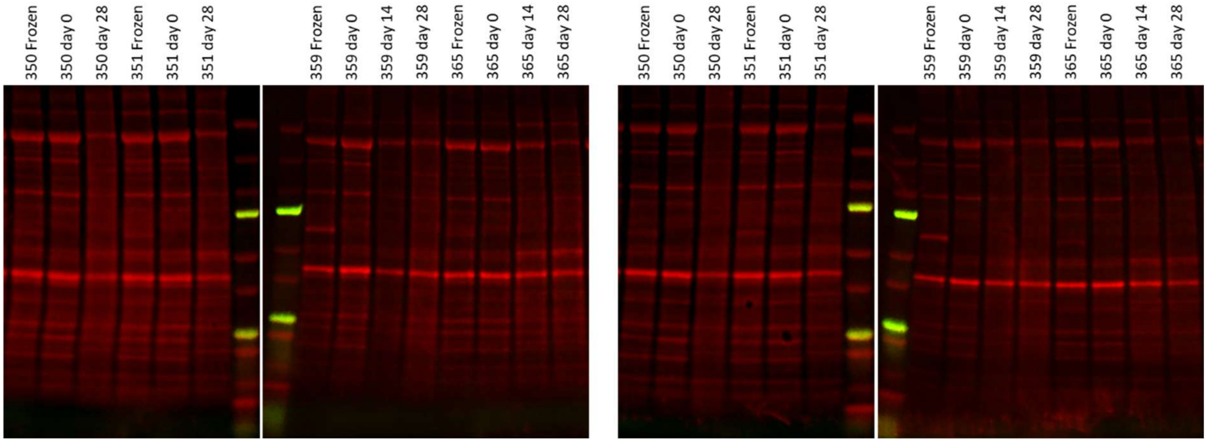


Total protein stain belonging to run 3.

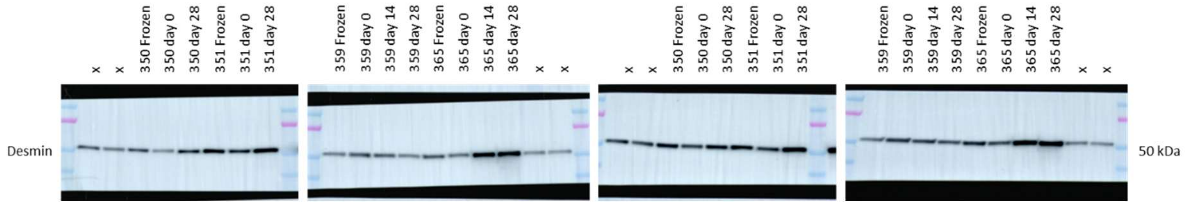


Uncropped western blots of run 4 corresponding to figure 6. Membranes were cut for imaging purposes only. Membranes were incubated with enhanced chemiluminescent (ECL) detection reagent (Amersham) and imaged using the Amersham Imager 600 (GE Healthcare Bio-Sciences AB). Protein expression was normalized against the total protein stain. X = samples that were not included in the analysis.

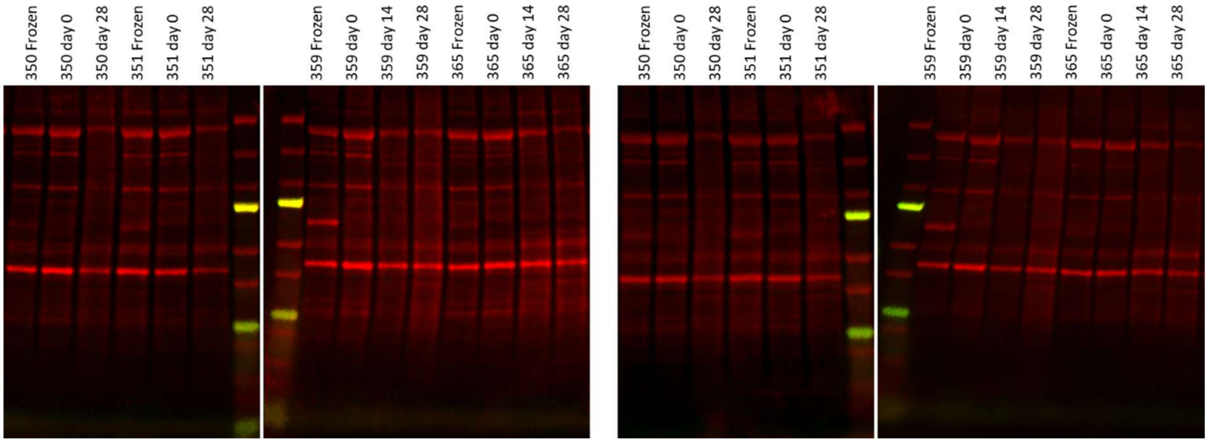


Total protein stain belonging to run 4.
